# HIV-1 Transmission during Early Infection in Men Who Have Sex with Men: A Phylodynamic Analysis

**DOI:** 10.1371/journal.pmed.1001568

**Published:** 2013-12-10

**Authors:** Erik M. Volz, Edward Ionides, Ethan O. Romero-Severson, Mary-Grace Brandt, Eve Mokotoff, James S. Koopman

**Affiliations:** 1 Department of Infectious Disease Epidemiology, Imperial College London, London, United Kingdom; 2 Department of Statistics, University of Michigan, Ann Arbor, Michigan, United States of America; 3 Theoretical Biology and Biophysics, Los Alamos National Laboratory, Los Alamos, New Mexico, United States of America; 4 Michigan Department of Community Health, Detroit, Michigan, United States of America; 5 Department of Epidemiology, University of Michigan, Ann Arbor, Michigan, United States of America; Imperial College London, London, United Kingdom

## Abstract

**Background:**

Conventional epidemiological surveillance of infectious diseases is focused on characterization of incident infections and estimation of the number of prevalent infections. Advances in methods for the analysis of the population-level genetic variation of viruses can potentially provide information about donors, not just recipients, of infection. Genetic sequences from many viruses are increasingly abundant, especially HIV, which is routinely sequenced for surveillance of drug resistance mutations. We conducted a phylodynamic analysis of HIV genetic sequence data and surveillance data from a US population of men who have sex with men (MSM) and estimated incidence and transmission rates by stage of infection.

**Methods and Findings:**

We analyzed 662 HIV-1 subtype B sequences collected between October 14, 2004, and February 24, 2012, from MSM in the Detroit metropolitan area, Michigan. These sequences were cross-referenced with a database of 30,200 patients diagnosed with HIV infection in the state of Michigan, which includes clinical information that is informative about the recency of infection at the time of diagnosis. These data were analyzed using recently developed population genetic methods that have enabled the estimation of transmission rates from the population-level genetic diversity of the virus. We found that genetic data are highly informative about HIV donors in ways that standard surveillance data are not. Genetic data are especially informative about the stage of infection of donors at the point of transmission. We estimate that 44.7% (95% CI, 42.2%–46.4%) of transmissions occur during the first year of infection.

**Conclusions:**

In this study, almost half of transmissions occurred within the first year of HIV infection in MSM. Our conclusions may be sensitive to un-modeled intra-host evolutionary dynamics, un-modeled sexual risk behavior, and uncertainty in the stage of infected hosts at the time of sampling. The intensity of transmission during early infection may have significance for public health interventions based on early treatment of newly diagnosed individuals.

*Please see later in the article for the Editors' Summary*

## Abstract 2

Erik Volz and colleagues use HIV genetic information from a cohort of men who have sex with men in Detroit, USA to dissect the timing of onward transmission during HIV infection.

*Please see later in the article for the Editors' Summary*

## Editors' Summary

**Background:**

Since the first recorded case of AIDS in 1981, the number of people infected with HIV, the virus that causes AIDS, has risen steadily. About 34 million people are currently HIV-positive, and about 2.5 million people become newly infected with HIV every year. Because HIV is usually transmitted through unprotected sex with an infected partner, individuals can reduce their risk of infection by abstaining from sex, by having only one or a few partners, and by always using condoms. Most people do not become ill immediately after infection with HIV, although some develop a short flu-like illness. The next stage of HIV infection, which may last more than ten years, also has no major symptoms, but during this stage, HIV slowly destroys immune system cells. Eventually, the immune system can no longer fight off infections by other disease-causing organisms, and HIV-positive people then develop one or more life-threatening AIDS-defining conditions, including unusual infections and specific types of cancer. HIV infection can be controlled, but not cured, by taking a daily cocktail of antiretroviral drugs.

**Why Was This Study Done?:**

The design of effective programs to prevent the spread of HIV/AIDS depends on knowing how HIV transmissibility varies over the course of HIV infection. Consider, for example, a prevention strategy that focuses on increasing treatment rates: antiretroviral drugs, in addition to reducing illness and death among HIV-positive people, reduce HIV transmission from HIV-positive individuals. “Treatment as prevention” can only block transmissions that occur after diagnosis and entry into care. However, the transmissibility of HIV per sexual contact depends on a person's viral load, which peaks during early HIV infection, when people are often unaware of their HIV status and may still be following the high-risk patterns of sexual behavior that caused their own infection. Epidemiological surveillance data (information on HIV infections within populations) can be used to estimate how many new HIV infections occur within a population annually (HIV incidence) and the proportion of the population that is HIV-positive (HIV prevalence), but cannot be used to estimate the timing of transmission events. In this study, the researchers use “phylodynamic analysis” to estimate HIV incidence and prevalence and the timing of HIV transmission during infection. HIV, like many other viruses, rapidly accumulates genetic changes. The timing of transmission influences the pattern of these changes. Viral phylodynamic analysis—the quantitative study of how epidemiological, immunological, and evolutionary processes shape viral phylogenies (evolutionary trees)—can therefore provide estimates of transmission dynamics.

**What Did the Researchers Do and Find?:**

The researchers obtained HIV sequence data (collected for routine surveillance of antiretroviral resistance mutations) and epidemiological surveillance data (including information on the stage of infection at diagnosis) for 662 HIV-positive men who have sex with men living in the Detroit metropolitan area of Michigan. They constructed a phylogenetic tree from the sequences using a “relaxed clock” approach and then fitted an epidemiological model (a mathematical model that represents the progress of individual patients through various stages of HIV infection) to the sequence data. Their approach, which integrates surveillance data and genetic data, yielded estimates of HIV incidence and prevalence among the study population similar to those obtained from surveillance data alone. However, it also provided information about HIV transmission that could not be obtained from surveillance data alone. In particular, it allowed the researchers to estimate that, in the current HIV epidemic among men who have sex with men in Detroit, 44.7% of HIV transmissions occur during the first year of infection.

**What Do These Findings Mean?:**

The robustness of these findings depends on the validity of the assumptions included in the researchers' population genetic model and on the accuracy of the data fed into the model, and may not be generalizable to other cities or to other risk groups. Nevertheless, the findings of this analysis, which can be repeated in any setting where HIV sequence data for individual patients can be linked to patient-specific clinical and behavioral information, have important implications for HIV control strategies based on the early treatment of newly diagnosed individuals. Because relatively few infected individuals are diagnosed during early HIV infection, when the HIV transmission rate is high, it is unlikely, suggest the researchers, that the “treatment as prevention” strategy will effectively control the spread of HIV unless there are very high rates of HIV testing and treatment.

**Additional Information:**

Please access these websites via the online version of this summary at http://dx.doi.org/10.1371/journal.pmed.1001568.

- This study is further discussed in a *PLOS Medicine* Perspective by Timothy Hallett
- Information is available from the US National Institute of Allergy and Infectious Diseases on HIV infection and AIDS
- NAM/aidsmap provides basic information about HIV/AIDS and summaries of recent research findings on HIV care and treatment
- Information is available from Avert, an international AIDS charity, on many aspects of HIV/AIDS, including information on HIV treatment as prevention (in English and Spanish)
- The *PLOS Medicine* Collection Investigating the Impact of Treatment on New HIV Infections provides more information about HIV treatment as prevention
- A *PLOS Computational Biology* Topic Page (a review article that is a published copy of record of a dynamic version of the article as found in Wikipedia) about viral phylodynamics is available
- The US National Institute of Health–funded HIV Sequence Database contains HIV sequences and tools to analyze these sequences
- Patient stories about living with HIV/AIDS are available through Avert; the charity website Healthtalkonline also provides personal stories about living with HIV

## Introduction

Variation in the timing of transmissions over the course of an infection can have large consequences for the design of HIV prevention programs [1]. For example, interventions focused on increasing treatment rates (treatment as prevention [TasP]) are only able to block transmissions that occur after diagnosis, entry into care, and consistent adherence to antiretroviral treatment. Consequently, TasP will preempt fewer transmissions as the proportion of transmissions from early infections rises. Highly elevated transmission rates early in infection are both biologically and sociologically plausible. The transmissibility of HIV per sexual encounter depends on the viral load within infected hosts [2],[3], which peaks during early HIV infection (EHI) and also rises during stage 3 (AIDS) [4]. Likewise, a recently infected individual is likely to have been infected during a period of high-risk behavior; if the high-risk behavior extends through EHI, then the interaction of elevated viral load and risky behavior can potentially elevate transmission rates during EHI.

Several mathematical analyses have argued that transmission rates during early infection drive the HIV-1 epidemic [5],[6]. That early infection transmissions drove the early epidemic is clear. But what drives transmission later in the epidemic remains controversial. A recent survey of estimates of the proportion of transmissions from EHI based on mathematical models of HIV transmission found very wide ranges, including scenarios where nearly all or almost no transmissions were coming from EHI [2]. The surveyed mathematical studies [2] attempted to fit diverse models of HIV transmission to indirect data such as diagnoses over time. The variation in parameter estimates could be due to real differences between the study populations, but it could also be due to a fundamental limitation of the data used to fit these kinds of models. Mathematical models of HIV transmission are essential for understanding HIV dynamics, but model-based analysis of diagnosis data alone has not yielded consistent estimates of the timing of HIV transmission over the course of infection.

Direct measurement of the timing of HIV transmission is possible in large population-based cohort studies that follow recently infected individuals and their uninfected partners [7],[8]. Careful monitoring for seroconversion of the uninfected partner and administration of questionnaires about sexual practices can paint an empirically driven picture of the timing of transmission events over the course of an infection. However, longitudinal partner studies are expensive and lack generalizability because risk behavior is highly variable over time and between risk groups.

Traditional surveillance data that are used to estimate incidence and prevalence of infection have little value for estimating the intensity of transmission during EHI, because outside of the very early epidemic period, a given incidence curve can be consistent with either high or low levels of EHI transmission. Methods have been developed to back calculate the incidence over time [9],[10] using information about the stage of infection of patients at the time of diagnosis, and estimates can be further refined by incorporating diverse data sources such as behavioral surveillance and seroprevalence surveys [11]. These methods are essential in evaluating the efficacy of prevention programs; however, the timing of transmission events cannot be identified from incidence data alone [12].

HIV genetic sequence data can potentially augment traditional surveillance data to estimate the timing of HIV transmissions. After the advent of highly effective antiretroviral therapy (HAART), increasing concerns about transmitted drug-resistant mutant strains of HIV, coupled with rapidly dropping prices for genetic sequencing, led to an abundance of HIV genetic sequence data from infected individuals in nearly all regions of the United States. There is substantial molecular epidemiological evidence that variation in transmission rates over the course of infection influences the genetic diversity of HIV [13]–[16]. For example, viral sequences isolated from patients who were recently infected tend to be phylogenetically clustered (more closely related to one another than expected by chance). Simple models of HIV transmission have been shown to reproduce these phylogenetic patterns [17], suggesting that the transmission rate from EHI could be identifiable from genetic data. The probability of observing a particular viral phylogeny depends not only on the historical dynamics of HIV in the population but also on the stage of each patient at the time of sampling. For example, a sample comprised of only EHI patients will yield a different phylogeny than one comprised of only AIDS patients, the former having many more short external branches [17].

Recent advances [18]–[21] in population genetic methods have enabled the fitting of formal epidemiological models to viral sequence data. We use these methods to estimate HIV incidence, HIV prevalence, and the timing of transmission using both genetic sequence data and conventional HIV surveillance data. These methods may detect intensified transmission during EHI and reduced transmission following diagnosis, and may illustrate how the fraction of transmissions attributable to EHI has varied over the course of the epidemic.

## Methods

### Ethics Statement

This research was reviewed by the Institutional Review Boards at the University of Michigan and the Michigan Department of Community Health (MDCH). Data used in this research were originally collected for HIV surveillance purposes. Data were anonymized by staff at the MDCH before being provided to investigators. Because this research falls under the original mandate for HIV surveillance and the data were de-identified, the study was classified as human subjects research but was exempt from further Institutional Review Board review.

### Data

As previously described in [17], the MDCH curates a database of partial pol HIV-1 sequences collected as part of routine clinical care and surveillance of drug-resistant mutant strains. MDCH provided an anonymized database of 9,002 sequences linked to clinical, demographic, and behavioral covariates of the patients from whom the sequences were isolated. Sequences were collected from October 14, 2004, through February 24, 2012 (Figure S1). 2,808 of these sequences correspond to men who have sex with men (MSM) in the Detroit metropolitan area (DMA), Michigan. To be included in the analysis each record must (1) have an HIV-1 subtype B sequence, (2) have a sequence from a HAART-naive patient within 12 mo of initial diagnosis, and (3) be collected from a man who has sex with men, residing in the DMA. Additionally, to achieve an analytically tractable sample size, we restricted our analyses to records that (4) have a high-quality sequence of at least 1,200 nucleotides. 662 of 2,808 sequence records collected from DMA MSM met all inclusion criteria. Demographic and clinical attributes of the study sample and the population are described in Table 1 and in Data S1. Details of the sequence selection, alignment, and quality control are contained in Text S1.

**Table 1:** Comparison of demographic and clinical variables for DMA MSM with sequences and those included in the estimated phylogenies.

Variable	Category	DMA MSM with Sequences		DMA MSM in Phylogeny	
		*n*	Percent	*n*	Percent
**Race**					
	Black	1,333	69%	473	71%
	White	497	26%	153	23%
	Hispanic	52	3%	24	4%
	Multiracial/unknown/other	55	2%	12	2%
**County**					
	Detroit	1,095	56%	359	54%
	Oakland	377	19%	140	21%
	Wayne	287	15%	88	13%
	Macomb	131	7%	61	10%
	St. Clair	21	1%	8	1%
	Monroe	16	1%	1	0%
	Lapeer	10	1%	5	1%
**AIDS** a		416	21%	121	18%
**FAS+** b		143	7%	88	13%

^a^ HIV diagnosis concurrent with AIDS diagnosis.

^b^ HIV diagnosis concurrent with low sequence ambiguity.

MDCH also provided anonymized surveillance data for 30,200 diagnoses reported in Michigan through March of 2012. 9,127 of these records corresponded to diagnoses from MSM in the DMA. These records contained CD4 cell counts, primary risk behavior, county of residence, and diagnosis dates of any AIDS-defining illnesses. The mean CD4 count upon diagnosis and number of AIDS/non-AIDS diagnoses were abstracted from these data and used for model-fitting and validation. Throughout this manuscript, “AIDS” refers to stage 3 HIV infection as defined in [4].

### Phylogenetic Inference

The pattern and timing of coalescent events were inferred using relaxed clock phylogenetic methods [22] implemented in BEAST [23]. We used a log-normal relaxed clock model informed by the sequence data [22] that accounts for variable evolutionary rates within [24] and between lineages. The full parameters of the BEAST analysis, convergence diagnostics, and details of sequence alignment and quality control are reported in Text S1 [25]–[29].

To give insight into how frequently HIV lineages are introduced into DMA MSM from other geographic or risk behavior groups, we supplemented our data with 100 sequences from the Los Alamos National Laboratory HIV Sequence Database. Sequences with high similarity to at least one of 662 DMA MSM sequences were sampled. These sequences were included in subsequent phylogenetic and coalescent analysis.

To ease the computational burden of analyzing 662 sequences, phylogenetic analysis proceeded in two steps. First a neighbor joining tree was calculated using all sequences (TN93+Gamma model). Then the tree was divided into nine clades by selecting branches close to the root of the tree. Nine disjoint multiple sequence alignments were generated, corresponding to taxa in each clade, which were then independently analyzed with BEAST. Ten trees were sampled from the results of these nine BEAST analyses (90 phylogenies in total) in order to capture uncertainty in topology and branch lengths. These trees were used in subsequent coalescent analysis.

We found that the estimate of the height of the tree—the time to the most recent common ancestor (TMRCA) of the whole sample—was not well identified by the sequence data alone. This common issue arises when the height of the tree and the mean evolutionary rate cannot be fully resolved. To alleviate this problem, we constrained the height of the tree by using a uniform prior on the TMRCA between 1970 and 1982. In subsequent coalescent analyses, we sampled the posterior distribution of trees estimated by BEAST and then merged nine clades at a polytomous root. To render branch lengths comparable between different BEAST analyses, we calculated the mean substitution rate across a sample of trees from each of nine BEAST analyses and then adjusted the substitution rate within each tree to this mean value. Because phylogenetic relationships in the distant past carry little information about the epidemic close to the present, and in order to reduce the computational burden of fitting the population genetic model, the subsequent coalescent analysis used only the portion of the tree dating from 1990 onwards.

### Transmission Model

**Figure 1:**
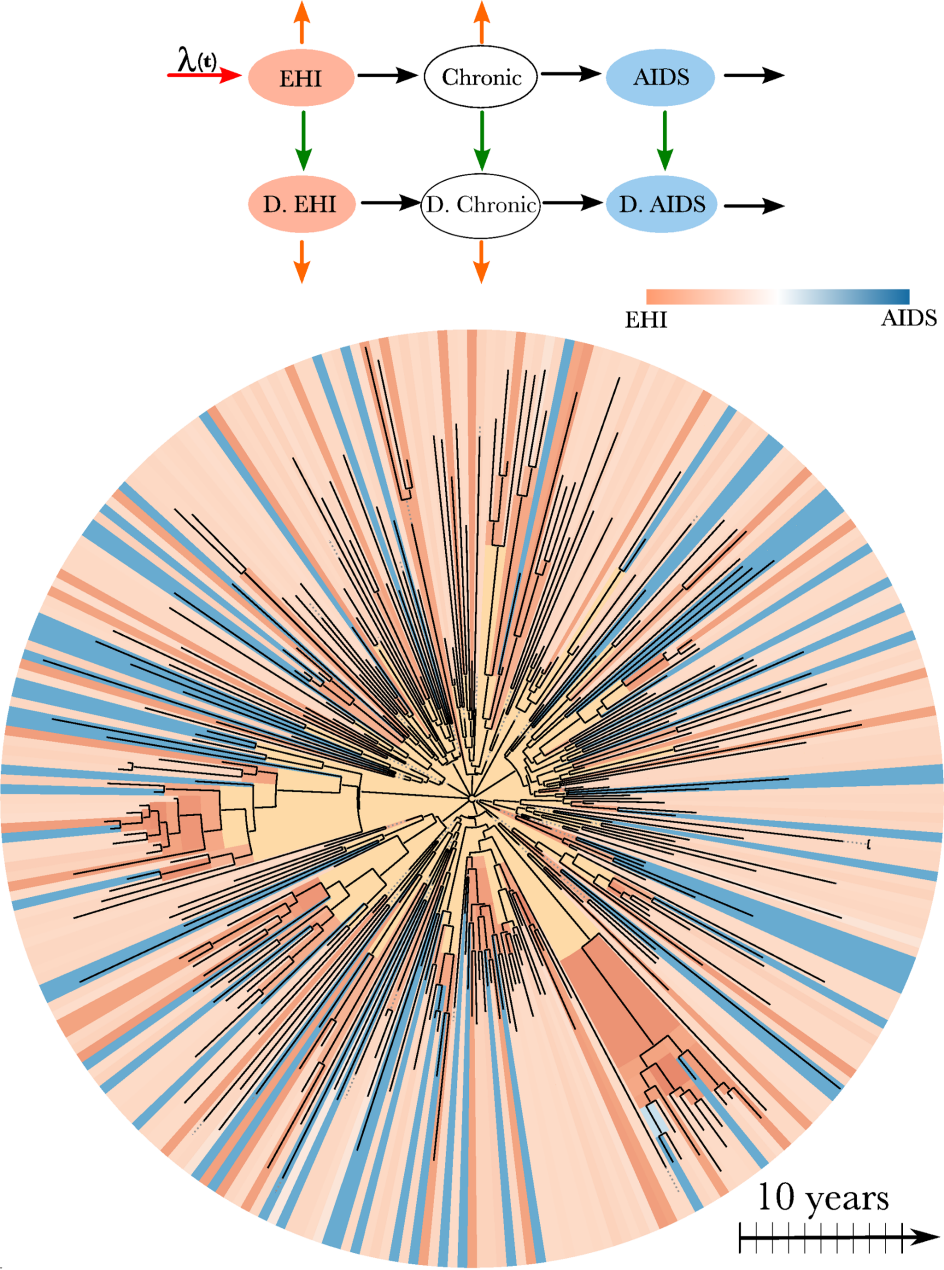
HIV transmission model and phylogeny. Top: A flow diagram describing the mathematical model fitted to surveillance time series and the HIV-1 phylogeny. Arrows of different colors represent the time-dependent rates at which transitions occur. Infected individuals progress from EHI to AIDS and may also become diagnosed (“D.”), as represented by black and green arrows. Orange arrows represent natural mortality. Incidence occurs at the rate λ(*t*) (red arrow). A more detailed diagram is shown in Figure S6. Bottom: HIV-1 phylogeny comprising virus samples from 662 patients and ancestral states estimated using the methods in [18]. The tree has been randomly downsampled to include 250 terminals for perspective. Colors at the terminals of the phylogeny represent the estimated stage of infection of the host at the time of sampling based on clinical data. Colors on the interior of the phylogeny represent the estimated stage of infection of the host harboring virus that is ancestral to the sample. Yellow corresponds to lineages that are likely to represent infections from outside of the DMA MSM risk group.

The transmission model that we used to estimate the incidence, prevalence, and timing of transmission events is an extension of a model used by Bezemer et al. [12] and Hogan et al. [30]. There are three essential components to the model structure: the incidence rate, the diagnosis rate, and the natural history of infection. The model is illustrated in Figure 1 and described in detail in Text S2. The natural history of infection is modeled with a system of ordinary differential equations that tracks infected individuals as they progress through EHI, three chronic stages of infection, and AIDS. Diagnosed and treated individuals progress through infection at a reduced rate. The model incorporates empirical death rates from natural and AIDS-related causes. The model closely reproduces empirical observations regarding the time from infection to AIDS (Text S2).

We defined EHI to have a mean duration of 1 y. This duration was chosen instead of the more commonly used durations of 2 or 6 mo because the simulation studies described in Text S3 revealed that transmission rates for shorter periods were not identifiable given the available number and quality of HIV sequences.

Both the diagnosis rate and incidence rate were modeled using separate cubic B-splines [30]. Splines are a semi-parametric method for defining a very wide range of curves with relatively few parameters. Using splines is a flexible approach that can approximately capture patterns generated by heterogeneities in behavior and diagnosis patterns without explicitly modeling them.

We also modeled importation of lineages into the DMA MSM risk group by adding an additional compartment that represents infected hosts outside of the DMA MSM risk group. DMA MSM emigrate out of the risk group at a constant per capita rate, and immigration rates balance emigration rates by design, such that prevalence is unchanged by migration dynamics.

We also estimated the HIV incidence from the surveillance data using a back-calculation method [9]. The back-calculation estimator is derived from sample survey statistics and is based on the ratio of the number of total diagnoses to the subset determined to have been recently infected [31].

### Parameter Estimation

The stage of infection at the time of sequencing of every patient with sampled sequence data was estimated using a naive Bayes classification method as described in “Estimating Stage of Infection” in Text S4. Covariates that were used for estimating stage of infection included the CD4 counts within 6 mo of diagnosis, whether the patient was diagnosed with AIDS within 2 mo of HIV diagnosis, and a measure of HIV sequence diversity. High sequence diversity is an indicator of a diverse intra-host viral population resulting from a long period of intra-host evolution and has previously been shown to be highly informative about the recency of infection [32]. The transmission model was fitted by maximum likelihood. The likelihood of the joint diagnosis time series data and of the genetic sequence data is given in Text S4.

Full derivations of the likelihood of the genetic data are given in [18],[20],[33]. These methods model each lineage in the viral phylogeny at each time point as corresponding to a single infected host. The model does not assume complete sampling and correctly accounts for the possibility that a lineage may pass through multiple unsampled hosts. Each node in the phylogeny is modeled as corresponding to a transmission event. These are reasonable approximations for many rapidly evolving RNA viruses including HIV [20],[34],[35] (see simulations in Text S3). The likelihood of the genetic data is computed by deriving the probability that each lineage in the phylogeny at each time point corresponds to an infected individual at a given stage of infection and diagnosis status. For example, the estimated stage of infection on the interior of the phylogeny is illustrated in Figure 1 for the HIV phylogeny of 662 patients. This figure shows that the state of an ancestral lineage at an internal node of the tree is likely to correspond to an EHI, owing to the large fraction of transmissions attributable to newly infected individuals.

The likelihood was numerically optimized using the simplex method via optim in R [36] to obtain maximum likelihood estimates (MLEs) of the transmission model parameters. Likelihood profiles were calculated for each transmission parameter. Credible intervals were calculated using an empirical Bayes approximation [37] (see “Model fitting” in Text S4). This approach uses a prior distribution that is calculated directly from the data. We constructed a multivariate uniform prior with bounds given by the 97.5% CIs calculated for each transmission parameter using the profile method.

To estimate parameters describing incidence, prevalence, and diagnosis rates through time, the model depicted in Figure 1 was fitted to diagnostic time series without using genetic data. To estimate the relative contribution of different stages of infection to total transmissions, the model was fitted to the genetic data while keeping fixed the parameters that describe incidence, prevalence, and diagnosis rates through time. This approach does not make full use of the genetic data, which may carry information about incidence and prevalence as well, but it is computationally efficient, since only a few parameters need to be estimated with the genetic data.

The robustness of inferences to phylogenetic error was assessed both by simulation techniques and by replicating parameter estimates across multiple independent estimated phylogenies. Uncertainty of the topology and branch lengths of estimated phylogenies (Figure S2) can lead to error in parameter estimates that is difficult to quantify. In “Simulations and Sensitivity Analysis” in Text S3, a simulation experiment is described that demonstrates that transmission rates in the first 6 mo of the infectious period cannot be inferred on the basis of the number and length of currently available sequences. Simulation experiments also show that it is feasible to estimate transmission rates within the first year (our definition of EHI) and to distinguish differing rates between chronic infection and late infection.

## Results

**Figure 2:**
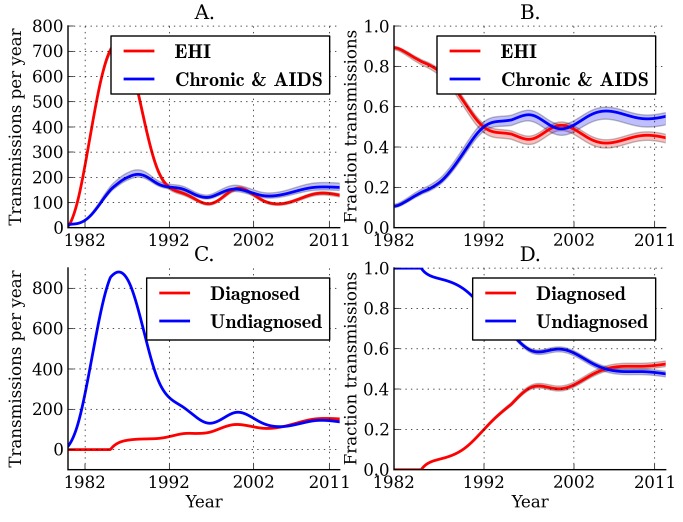
Estimated transmission patterns through time. Lines show the MLE, and shaded regions show the 95% credible interval. (A) Estimated number of transmissions originating from individuals in different stages of infection: EHI, chronic infection, and AIDS. (B) The estimated fraction of transmissions attributable to EHI, chronic infection, and AIDS. (C) Estimated number of transmissions originating from diagnosed and undiagnosed individuals through time. (D) The estimated fraction of transmissions attributable to diagnosed and undiagnosed infections through time. Estimated credible intervals reflect the fit of a single model to the data and do not incorporate uncertainty due to model misspecification error.

Figure 2A and 2B illustrates the estimated fractions and total numbers of transmissions that originated from EHI and chronic infections. Transmissions by EHI dominated during the early epidemic (1980–1990), reflecting the larger prevalence of EHI as a proportion of the total number of infections and the relative intensity of transmission from this group [6]. The estimated fraction of transmissions attributable to EHI has stabilized since the early 1990s. Combined analysis of genetic and time series data yields an estimate of 44.7% (95% CI, 42.2%–46.4%) for the fraction of transmissions originating from EHI (approximately the first year of infection) at the beginning of 2007. This reflects about a 4.2-fold increase in transmission rates during EHI relative to the entire infectious period and an 8-fold increase in transmission rates relative to chronic infection. This credible interval and subsequent credible intervals are based on the fitting of a single model. These estimates do not reflect uncertainty due to model misspecification. Models with different parameterizations for the infectiousness of diagnosed individuals or different interaction effects could yield different estimates (Table S1).

**Figure 3:**
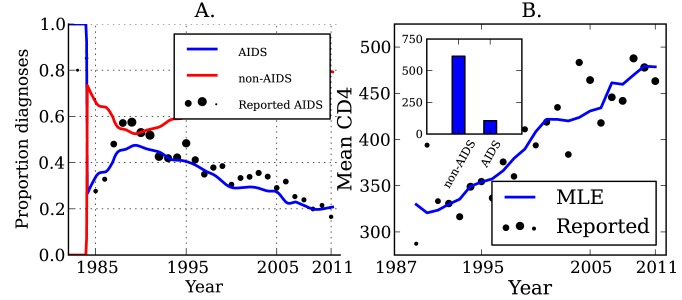
HIV diagnoses and CD4 cell count through time. (A) The sample proportion (points) and estimated proportion (lines) of diagnoses that are concurrent with AIDS diagnosis over time, and the estimated proportion of diagnoses that are not concurrent with AIDS. The diameter of points is proportional to the number of diagnoses used to calculate the proportions. (B) The sample mean (points) and estimated mean (lines) CD4 cell count in newly diagnosed individuals over time. The mean is calculated from CD4 counts aggregated by year. The diameter of points is proportional to the number of CD4 counts used to calculate the mean. Inset: The mean CD4 cell count by stage of infection, which gives the best fit (least squares) to the observed trend in mean CD4 count over time.

The true number of transmissions from diagnosed individuals depends on many factors, including the number of infected individuals who are diagnosed, the extent to which knowledge of infection reduces infectiousness, and the effectiveness of HAART at reducing transmission probabilities per sexual act. We estimate that transmissions from diagnosed individuals have trended upwards in recent years, which reflects that a steadily increasing proportion of infections are now diagnosed (Figure 2). We estimate that 52.4% (95% CI, 51.1%–53.9%) of transmissions originated from diagnosed individuals in 2007 (Figure 2C and 2D) [38]. We found that the proportion of transmissions from EHI mirrors the trend in diagnoses late in infection over time. Figure 3 shows the fraction of diagnoses concurrent with AIDS diagnosis and the estimated number of diagnoses during EHI. We categorized all diagnoses prior to the availability of the first HIV test (1985) as AIDS. AIDS diagnoses have fallen over time, while the number of diagnoses during EHI has risen dramatically since the mid-1990s from zero to an estimated 21.7% of current diagnoses. Estimated diagnosis rates are shown in Figure S3. The reported number of diagnoses that are concurrent with AIDS diagnosis is in close agreement with the model-estimated number of AIDS diagnoses in Figure 3. We found that diagnosis rates have risen consistently since the HIV test became available in 1985, and the time from infection to diagnosis has steadily decreased.

The mean CD4 cell count of patients at the time of diagnosis is indicative of the trend of increasing diagnosis rates. The average CD4 count for new diagnoses by year is shown in Figure 3. While CD4 counts are a very noisy and unreliable indicator of time since infection on an individual basis, aggregated CD4 counts follow an almost linear trend. The CD4 data were not used when estimating diagnosis rates, but are a useful check that the model is giving realistic estimates. Also shown in Figure 3 is the mean CD4 count for new diagnoses predicted by the MLE model fit, as well as the best-fitting CD4 counts by stage of infection as described in “CD4 and Model Validation” in Text S3.

**Figure 4:**
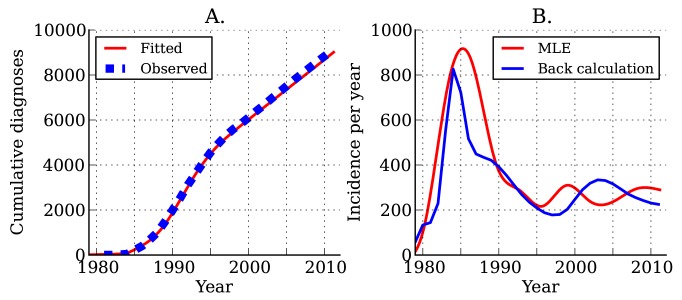
Estimated HIV diagnoses and HIV incidence through time. (A) Actual (blue) and estimated (red) cumulative HIV diagnoses in DMA MSM. (B) Estimated incidence of infection over time. The red line shows estimated incidence from surveillance time series data. The blue line shows estimated incidence using the back-calculation method.

Estimated incidence and cumulative diagnoses of HIV infection for DMA MSM are shown in Figure 4. As of the beginning of 2012, there have been a total of 9,127 HIV diagnoses in DMA MSM. We estimate that there have been a total of 12,139 infections in DMA MSM, of which 6,084 are still living; of these 6,084, we estimate that 5,233 have been diagnosed. The estimated current number of prevalent infections exceeds the number of living diagnosed individuals by 16.3%. Approximately one in seven infected individuals is unaware of his infection [39]. Estimates using the HIV model are very similar to those obtained from widely used back-calculation methods [10],[31] (Figure 4B), despite the fact that the HIV model uses much more data and more realistically models diagnosis rates and the natural history of infection.

Following the initial rapid rises in the 1980s, we found that incidence (Figure 4B) and the number of undiagnosed infections (Figure S4) have been steady since the mid-1990s. The total number of individuals living with diagnosed infections has trended upwards since 1995 because of reduced mortality with effective treatment (Figure S4). We estimate that incidence at the beginning of 2007 stood at 256 new infections per year, and incidence at the beginning of 2012 stood at 290 new infections per year; however, there is a great deal of uncertainty in estimated incidence close to the present.

Infectiousness can vary with stage of infection and diagnosis status; however, it is not clear a priori which factors are necessary to include in a model to provide a satisfactory fit to the data. We conducted a data-driven comparison of several models that differed in how relative infectiousness was described. Results of this analysis are shown in Table S1. In the most general model, three parameters describe the relative infectiousness of diagnosed individuals, those with chronic infection, and those with AIDS. The simplest models include only one parameter describing the relative infectiousness of those with late infection (chronic and AIDS) or diagnosed individuals. Analyzing the fits of these models to the phylogenetic data provides strong support for the two-parameter model, which includes parameters only for infectiousness of chronic infection and AIDS. Thus, we do not detect reduced infectiousness with diagnosis; however, this finding may be related to sample selection. Only sequences from recently diagnosed individuals were considered in this analysis. It is possible that analysis of sequences from individuals who have been diagnosed for a long time may reveal reduced infectiousness with diagnosis and treatment; however, the present HIV model is not equipped to fit such data. The estimated fractions of transmissions from EHI and diagnosed individuals are consistent across model variants, with the exception of one model that fit the data poorly.

To give greater intuition into why variation in transmission rates is identifiable from genetic data, we present simulated trees in “Simulated Trees” in Text S3. Simulations were carried out under scenarios in which individuals with EHI transmit at greater rates than those with chronic infections or at equal rates. The times and states of patients in the simulated trees were chosen to match the real data. In addition to the results presented in [17], these simulations give a graphical representation of how EHI transmission influences HIV phylogenetic structure.

## Discussion

We have estimated that almost half of transmissions occur within the first year of infection in the contemporary HIV epidemic among MSM in the DMA. This inference was made possible by recently developed population genetic methods [18],[20],[33] that enable characterization of the major sources of transmission. These findings may have significance for control strategies based on prophylactic use of antiretroviral medications. TasP [1],[40] is a strategy based on early administration of HAART following HIV diagnosis in an effort to suppress viral loads and reduce transmission probabilities. The impact that transmission during early infection will have on TasP strategies has been a subject of recent debate. Cohen et al. [41] present contrasting views regarding the potential impact of EHI transmission on TasP effectiveness. A simplistic interpretation of our results would hold that a lower fraction of transmissions will be prevented by TasP because a large fraction of transmissions are likely to occur before diagnosis. A more nuanced view presented by Cohen et al. [41] holds that high transmission during EHI and observed incidence trends are consistent with a low reproduction number, and therefore TasP may nevertheless have large population-level impacts even if it prevents few transmissions directly. Contact patterns and fluctuations in risk behavior can markedly raise the fraction of transmissions from early infection [42], and these factors were not modeled in this study. These same factors may yield lower reproduction numbers at any given endemic prevalence level, so that TasP would have to prevent only a small fraction of all transmissions in order to have very large population effects.

The robustness of our conclusions depends on the sensitivity of the conclusions to potential violations of several assumptions on which the population genetic model is based. The population genetic model accounts for the effects of incomplete sampling and the possibility that a lineage in the viral phylogeny may pass through more than one infected host. However, the approach relies on the assumption that a single lineage at a given time corresponds to a single infected host. This assumption would be problematic if multiple lineages circulate in a host and are independently transmitted. But a growing body of evidence suggests that new infections are established by a very small number of viral particles [34],[43],[44]. Virus derived from a single transmitting host at a single transmission event is likely to have limited diversity. In contrast, dual infection from distinct partners may present a greater challenge to attempts to reconstruct epidemiological dynamics from genetic data. Virus derived from distinct partners is likely to be more diverse, which in the presence of high levels of recombination makes estimation of an accurate phylogeny difficult. Recent studies [45],[46] have found low prevalence of HIV dual infections (including super- and coinfection [47]). There is some evidence based on African heterosexual cohorts that the incidence of super-infection is comparable to the incidence of infection generally [48]. However, bias due to dual infection will depend on when transmission occurs during the course of infection. If transmission occurs early, it is more likely to occur before a host is multiply infected. Additionally, if the super-infecting strain has low abundance within the host, it is unlikely to have a large influence on phylogenies estimated from consensus sequences. We removed all sequences with evidence of recombination, further reducing the possibility of bias from dual-infected hosts. In “Dual Infection” in Text S3, we provide a simple calculation that gives the approximate bias that can be expected if the prevalence of super-infection is 10%. In this scenario, the estimated HIV incidence would be biased downwards by at most 3.75%.

This analysis assumes that the internal nodes of the phylogeny represent transmission events. In reality, the viral lineage that is transmitted may have arisen in the host some time before transmission occurred [35]. The magnitude of the potential bias introduced by this assumption is an empirical question that we have addressed by simulation in “Simulations and Sensitivity Analysis” in Text S3. By incorporating an empirical distribution [49],[50] for the time of common ancestry within hosts into epidemic simulations, we have established that intra-host evolutionary dynamics are unlikely to introduce large bias into our estimates.

The methods that we presented in this paper are sensitive to uncertainty both in the phylogeny and in the stage of infection of patients at the time of sampling. Estimation of stage of infection is very imprecise and is limited by the available clinical and self-reported data. Improved antibody avidity assays promise to greatly improve our ability to determine when newly diagnosed individuals were infected. Serial sampling with deep sequencing of the virus within hosts is another promising strategy to estimate the time since infection [51]. Increasing the number, length, and quality of sequence data can greatly improve the quality of the phylogenetic reconstruction. In this study, we were limited to using only about a quarter of potentially informative sequences by the computational demands of both estimating a relaxed clock phylogeny and fitting complex models to the estimated phylogeny (see “Computation” in Text S4). The value of genetic data for epidemiological inference will increase as computational techniques are developed that allow for the incorporation of more sequences.

The accuracy of the phylogenetic reconstruction may also be affected by sampling from different stages of infection. In our analysis, the sample was skewed towards viral sequences from EHI or late infections. The mean substitution rate varies over the course of infection [24], adding an extra layer of complexity to the phylogenetic analysis. We found that the mean substitution rate in external branches of the phylogeny is significantly correlated (Pearson correlation −0.17, *p*<0.001) with the estimated stage of infection of the patient from whom the virus was sampled (Figure S5). This correlation suggests that the relaxed clock methods that we used to estimate the branch lengths in units of time was flexible enough to account for variable rates of evolution over the course of infection.

While our conclusions are sensitive to many different sources of error, we can evaluate the robustness of our conclusions by reestimating parameters with simulated data where the true parameter values are known. In Text S3, we describe several simulation experiments designed to test the robustness of our estimates to error in the phylogeny and to errors arising from stochastic population dynamics. These simulations demonstrate that it is feasible to estimate EHI transmission rates given the available data. This analysis can be repeated with different HIV transmission models, which may be appropriate when more is known about heterogeneous risk behaviors and sexual networks. Sexual network heterogeneity influences HIV phylogenetic structure [52],[53], which may make it possible to estimate features of the sexual network from phylogenetic data [54]. Our estimated credible intervals are based on the fit of a single model to the data, and models that more realistically account for individual-level heterogeneities may yield different estimates. Although we report estimates based on the model that best fit the data, our estimated credible intervals do not account for error due to model misspecification.

The analysis we have presented can be replicated for other cities and risk groups where drug-resistant mutant strain sequence databases are available and can be linked to clinical and behavioral covariates for each patient. Phylodynamic analysis of HIV can supplement routine surveillance, addressing the need to monitor sources of transmission and generating the evidence necessary to efficiently allocate resources and assess control program effectiveness.

## Supporting Information

Data S1 **Comparison of clinical and demographic characteristics of patients selected for phylogenetic analysis and all patients diagnosed, 2004–2012.**
(TXT)

Figure S1 **Number of HIV sequences sampled in Michigan by year.**
(PNG)

Figure S2 **Comparison of estimated terminal branch lengths from relaxed clock phylogeny and the true branch lengths from a simulated tree.** Color indicates the stage of infection of patient at time of sampling. Darker colors indicate patients sampled earlier in the infectious period.
(PNG)

Figure S3 **Estimated diagnosis rates over time.**
(TIFF)

Figure S4 **Estimated prevalence of infection over time.**
(TIFF)

Figure S5 **HIV nucleotide substitution rate and stage of infection.** Blue points: the mean substitution rate is compared to the estimated stage of infection. The substitution rate for each patient corresponds to an external branch in the relaxed clock phylogeny estimated with BEAST. The stage of infection is estimated from AIDS-defining illness, the frequency of ambiguous sites of the HIV sequence, and CD4 as described in “Estimating Stage of Infection” in Text S4. Red line: linear regression.
(PNG)

Figure S6 **A flow diagram representing transitions made by infected individuals in the HIV model.** Boxes represent categories of individuals who are infected with HIV and who may be diagnosed or undiagnosed in any of five stages of infection. Arrows represent the time-varying rates with which individuals transition between categories.
(PNG)

Figure S7 **A schema illustrating how data were used at each stage of the analysis and how each analysis method was used to generate each result.** The corresponding supporting text file that discusses each method is also listed. Primary data are shown in the red rectangle, procedures are shown in yellow ellipses, intermediate results are shown in grey rectangles, and final results are shown in blue rectangles.
(PDF)

Figure S8 **A flow diagram representing transitions made by infected individuals in the source–sink HIV model.** Boxes represent categories of individuals who are infected with HIV and who may be diagnosed or undiagnosed in any of five stages of infection. Arrows represent the time-varying rates with which individuals transition between categories. The source and sink compartments represent migration of viral lineages in and out of the Detroit MSM risk group.
(PDF)

Figure S9 **Simulated durations of chronic infection, which we define as the interval from the end of early HIV infection to the beginning of AIDS.** Data are simulated based on data from the Multicenter AIDS Cohort Study.
(PDF)

Figure S10 **Antiretroviral uptake and usage through time.** Left: Date of diagnosis and self-reported date of first antiretroviral therapy usage for participants in the Medical Monitoring Project in Michigan. Instances in which first antiretroviral therapy usage precedes diagnosis may be due to self-administered prophylaxis or due to reporting error. Right: The estimated HAART availability, as a function of time.
(PNG)

Figure S11 **CD4 cell count by stage of infection at time of diagnosis.** Left: A linear regression fit to the distribution of root CD4 counts for EHI and AIDS. Right: The distribution of root CD4 counts for EHI and AIDS.
(PDF)

Figure S12 **The probability that a sequence originated from a patient with early HIV infection if the number of ambiguous sites is less than the given threshold (positive predictive value).**
(PNG)

Figure S13 **Likelihood surface for simulation experiment with demographic stochasticity.** Left: The coalescent likelihood versus β_c_ and δ. Right: Contour plot of the coalescent likelihood. The innermost contour shows all points within two log units of the maximum of the likelihood surface. The black circle indicates the true parameter value corresponding to the MLE in the main text. The black star indicates the maximum of the likelihood in the simulation experiment.
(PNG)

Figure S14 **A flow-chart representation of the simulation experiment to determine the robustness of inferences to phylogenetic error.**
(PDF)

Figure S15 **Estimated posteriors for the parameters β_1_ and β_25_ based on three trees estimated independently from BEAST.** Also shown are estimates based on the true coalescent tree, and the parameters used to generate the coalescent tree (red star).
(PNG)

Figure S16 **Within host coalescence of HIV lineages.** Left: Histogram of intra-host coalescent times (TMRCA) for all pairs of isochronously sampled sequences in [49]. Right: Time to seroconversion versus intra-host coalescent times for all pairs of isochronously sampled sequences for nine patients in [49]. The blue line shows the median TMRCA at each sample point, and the green line shows a linear regression of TMRCA on time since seroconversion.
(PDF)

Figure S17 **Likelihood surface for simulation experiment with intra-host evolution.** Left: The coalescent likelihood versus β_c_ and δ for a simulated tree where nodes correspond to intra-host coalescent events rather than transmission events. Right: Contour plot of the coalescent likelihood. The innermost contour shows all points within two log units of the maximum of the likelihood surface. The black circle indicates the true parameter value corresponding to the MLE in the main text. The black star indicates the maximum of the likelihood in the simulation experiment.
(PNG)

Figure S18 **Simulated coalescent trees for HIV models.** (A) A simulated coalescent tree under a scenario where individuals with EHI transmit at a rate equal to that of individuals with chronic infection or AIDS. (B) The HIV-1 phylogeny of 437 patients. (C) A simulated coalescent tree such that individuals with EHI transmit at a greater rate than those with chronic infection, as described by the MLE model fit in the main text. Terminals of the tree are colored according to stage of infection of the patient at the time of sampling. Red indicates those sampled during EHI or chronic infection stages. Blue indicates sampling during AIDS.
(PDF)

Table S1 **Model comparisons.**
(PDF)

Text S1 **Detailed methods for phylogenetic analysis.**
(PDF)

Text S2 **Detailed description of HIV transmission model.**
(PDF)

Text S3 **Model validation and simulations.**
(PDF)

Text S4 **Detailed methods used for model fitting and parameter estimation.**
(PDF)

## Abbreviations

DMA: Detroit metropolitan area
EHI: early HIV infection
HAART: highly effective antiretroviral therapy
MDCH: Michigan Department of Community Health
MLE: maximum likelihood estimate
MSM: men who have sex with men
TasP: treatment as prevention
TMRCA: time to the most recent common ancestor

## References

[1] Cohen M, Chen Y, McCauley M, Gamble T, Hosseinipour M, et al (2011) Prevention of HIV-1 infection with early antiretroviral therapy. N Engl J Med 365: 493–505. 21767103 10.1056/NEJMoa1105243 PMC3200068

[2] Cohen M, Shaw G, McMichael A, Haynes B (2011) Acute HIV-1 infection. N Engl J Med 364: 1943–1954. 21591946 10.1056/NEJMra1011874 PMC3771113

[3] Pilcher C, Tien H, Eron J Jr, Vernazza P, Leu S, et al (2004) Brief but efficient: acute HIV infection and the sexual transmission of HIV. J Infect Dis 189: 1785–1792. 15122514 10.1086/386333

[4] Schneider E, Whitmore S, Glynn K, Dominguez K, Mitsch A, et al (2008) Revised surveillance case definitions for HIV infection among adults, adolescents, and children aged <18 months and for HIV infection and AIDS among children aged 18 months to <13 years—United States, 2008. MMWR Recomm Rep 57: 1–12. 19052530

[5] Powers K, Ghani A, Miller W, Hoffman I, Pettifor A, et al (2011) The role of acute and early HIV infection in the spread of HIV and implications for transmission prevention strategies in Lilongwe, Malawi: a modelling study. Lancet 378: 256–268. 21684591 10.1016/S0140-6736(11)60842-8 PMC3274419

[6] Jacquez JA, Koopman JS, Simon CP, Longini IM Jr (1994) Role of the primary infection in epidemics of HIV infection in gay cohorts. J Acquir Immune Defic Syndr 7: 1169–1184. 7932084

[7] Baeten J, Overbaugh J (2003) Measuring the infectiousness of persons with HIV-1: opportunities for preventing sexual HIV-1 transmission. Curr HIV Res 1: 69–89. 15043213 10.2174/1570162033352110

[8] Gray R, Wawer M, Brookmeyer R, Sewankambo N, Serwadda D, et al (2001) Probability of HIV-1 transmission per coital act in monogamous, heterosexual, HIV-1-discordant couples in Rakai, Uganda. Lancet 357: 1149–1153. 11323041 10.1016/S0140-6736(00)04331-2

[9] Karon J, Song R, Brookmeyer R, Kaplan E, Hall H (2008) Estimating HIV incidence in the United States from HIV/AIDS surveillance data and biomarker HIV test results. Stat Med 27: 4617–4633. 18833636 10.1002/sim.3144

[10] Prejean J, Song R, Hernandez A, Ziebell R, Green T, et al (2011) Estimated HIV incidence in the United States, 2006–2009. PLoS ONE 6: e17502 doi:10.1371/journal.pone.0017502 21826193 10.1371/journal.pone.0017502 PMC3149556

[11] van Veen MG, Presanis AM, Conti S, Xiridou M, Stengaard AR, et al (2011) National estimate of HIV prevalence in the Netherlands: comparison and applicability of different estimation tools. AIDS 25: 229–237. 21150562 10.1097/QAD.0b013e32834171bc

[12] Bezemer D, de Wolf F, Boerlijst M, van Sighem A, Hollingsworth T, et al (2010) 27 years of the HIV epidemic amongst men having sex with men in the Netherlands: an in depth mathematical model-based analysis. Epidemics 2: 66–79. 21352777 10.1016/j.epidem.2010.04.001

[13] Pao D, Fisher M, Hué S, Dean G, Murphy G, et al (2005) Transmission of HIV-1 during primary infection: relationship to sexual risk and sexually transmitted infections. AIDS 19: 85–90. 15627037 10.1097/00002030-200501030-00010

[14] Yerly S, Junier T, Gayet-Ageron A, Amari E, von Wyl V, et al (2009) The impact of transmission clusters on primary drug resistance in newly diagnosed HIV-1 infection. AIDS 23: 1415–1423. 19487906 10.1097/QAD.0b013e32832d40ad

[15] Cuevas M, Muñoz-Nieto M, Thomson M, Delgado E, Iribarren J, et al (2009) HIV-1 transmission cluster with T215D revertant mutation among newly diagnosed patients from the Basque Country, Spain. J Acquir Immune Defic Syndr 51: 99–103. 19282784 10.1097/QAI.0b013e318199063e

[16] Aldous J, Pond S, Poon A, Jain S, Qin H, et al (2012) Characterizing HIV transmission networks across the United States. Clin Infect Dis 55: 1135–1143. 22784872 10.1093/cid/cis612 PMC3529609

[17] Volz E, Koopman J, Ward M, Brown A, Frost S (2012) Simple epidemiological dynamics explain phylogenetic clustering of HIV from patients with recent infection. PLoS Comput Biol 8: e1002552 doi:10.1371/journal.pcbi.1002552 22761556 10.1371/journal.pcbi.1002552 PMC3386305

[18] Volz E (2012) Complex population dynamics and the coalescent under neutrality. Genetics 190: 187–201. 22042576 10.1534/genetics.111.134627 PMC3249372

[19] Frost S, Volz E (2010) Viral phylodynamics and the search for an ‘effective number of infections’. Philos Trans R Soc Lond B Biol Sci 365: 1879–1890. 20478883 10.1098/rstb.2010.0060 PMC2880113

[20] Volz E, Pond S, Ward M, Leigh Brown A, Frost S (2009) Phylodynamics of infectious disease epidemics. Genetics 183: 1421–1430. 19797047 10.1534/genetics.109.106021 PMC2787429

[21] Stadler T, Kouyos R, von Wyl V, Yerly S, Böni J, et al (2012) Estimating the basic reproductive number from viral sequence data. Mol Biol Evol 29: 347–357. 21890480 10.1093/molbev/msr217

[22] Drummond A, Ho S, Phillips M, Rambaut A (2006) Relaxed phylogenetics and dating with confidence. PLoS Biol 4: e88 doi:10.1371/journal.pbio.0040088 16683862 10.1371/journal.pbio.0040088 PMC1395354

[23] Drummond A, Rambaut A (2007) BEAST: Bayesian evolutionary analysis by sampling trees. BMC Evol Biol 7: 214. 17996036 10.1186/1471-2148-7-214 PMC2247476

[24] Lee H, Perelson A, Park S, Leitner T (2008) Dynamic correlation between intrahost HIV-1 quasispecies evolution and disease progression. PLoS Comput Biol 4: e1000240 doi:10.1371/journal.pcbi.1000240 19079613 10.1371/journal.pcbi.1000240 PMC2602878

[25] Rambaut A (2011). Path-O-Gen [computer program]. Available: http://tree.bio.ed.ac.uk/software/pathogen/. Accessed 4 November 2013.

[26] Pond S, Posada D, Stawiski E, Chappey C, Poon A, et al (2009) An evolutionary model-based algorithm for accurate phylogenetic breakpoint mapping and subtype prediction in HIV-1. PLoS Comput Biol 5: e1000581 doi:10.1371/journal.pcbi.1000581 19956739 10.1371/journal.pcbi.1000581 PMC2776870

[27] Bennett D, Camacho R, Otelea D, Kuritzkes D, Fleury H, et al (2009) Drug resistance mutations for surveillance of transmitted HIV-1 drug-resistance: 2009 update. PLoS ONE 4: e4724 doi:10.1371/journal.pone.0004724 19266092 10.1371/journal.pone.0004724 PMC2648874

[28] Pond S, Muse S (2005) Hyphy: hypothesis testing using phylogenies. In: Nielsen R, editor. Statistical methods in molecular evolution. pp. 125–181.

[29] Los Alamos National Laboratory (2011) Quality control: HIV-1 sequence quality analysis. Available: http://www.hiv.lanl.gov/content/sequence/QC/index.html. Accessed 4 November 2013.

[30] Hogan D, Zaslavsky A, Hammitt J, Salomon J (2010) Flexible epidemiological model for estimates and short-term projections in generalised HIV/AIDS epidemics. Sex Transm Infect 86: ii84–ii92. 21106520 10.1136/sti.2010.045104 PMC3173822

[31] Yan P, Zhang F, Wand H (2011) Using HIV diagnostic data to estimate HIV incidence: method and simulation. Stat Commun Infect Dis 3: 1–28.

[32] Kouyos R, von Wyl V, Yerly S, Böni J, Rieder P, et al (2011) Ambiguous nucleotide calls from population-based sequencing of HIV-1 are a marker for viral diversity and the age of infection. Clin Infect Dis 52: 532–539. 21220770 10.1093/cid/ciq164 PMC3060900

[33] Rasmussen D, Ratmann O, Koelle K (2011) Inference for nonlinear epidemiological models using genealogies and time series. PLoS Comput Biol 7: e1002136 doi:10.1371/journal.pcbi.1002136 21901082 10.1371/journal.pcbi.1002136 PMC3161897

[34] Zhu T, Mo H, Wang N, Nam D, Cao Y, et al (1993) Genotypic and phenotypic characterization of HIV-1 patients with primary infection. Science 261: 1179–1181. 8356453 10.1126/science.8356453

[35] Leitner T, Albert J (1999) The molecular clock of HIV-1 unveiled through analysis of a known transmission history. Proc Natl Acad Sci U S A 96: 10752–10757. 10485898 10.1073/pnas.96.19.10752 PMC17955

[36] R Core Team (2012) R: a language and environment for statistical computing. R Vienna: Foundation for Statistical Computing. Available: http://www.R-project.org/. Accessed 4 November 2013.

[37] Efron B (2010) Large-scale inference: empirical Bayes methods for estimation, testing and prediction. Cambridge: Cambridge University Press.

[38] Marks G, Crepaz N, Janssen R (2006) Estimating sexual transmission of HIV from persons aware and unaware that they are infected with the virus in the USA. AIDS 20: 1447–1450. 16791020 10.1097/01.aids.0000233579.79714.8d

[39] Campsmith M, Rhodes P, Hall H, Green T (2010) Undiagnosed HIV prevalence among adults and adolescents in the United States at the end of 2006. J Acquir Immune Defic Syndr 53: 619–624. 19838124 10.1097/QAI.0b013e3181bf1c45

[40] Montaner JS (2013) Treatment as prevention: toward an AIDS-free generation. Top Antivir Med 21: 110–114. 23981598 PMC6148874

[41] Cohen MS, Dye C, Fraser C, Miller WC, Powers KA, et al (2012) HIV treatment as prevention: debate and commentary—will early infection compromise treatment-as-prevention strategies? PLoS Med 9: e1001232 doi:10.1371/journal.pmed.1001232 22802728 10.1371/journal.pmed.1001232 PMC3393667

[42] Zhang X, Zhong L, Romero-Severson E, Alam SJ, Henry CJ, et al (2012) Episodic HIV risk behavior can greatly amplify HIV prevalence and the fraction of transmissions from acute HIV infection. Stat Commun Infect Dis 4: 1041. 24058722 10.1515/1948-4690.1041 PMC3778933

[43] Li H, Bar K, Wang S, Decker J, Chen Y, et al (2010) High multiplicity infection by HIV-1 in men who have sex with men. PLoS Pathog 6: e1000890 doi:10.1371/journal.ppat.1000890 20485520 10.1371/journal.ppat.1000890 PMC2869329

[44] Fischer W, Ganusov V, Giorgi E, Hraber P, Keele B, et al (2010) Transmission of single HIV-1 genomes and dynamics of early immune escape revealed by ultra-deep sequencing. PLoS ONE 5: e12303 doi:10.1371/journal.pone.0012303 20808830 10.1371/journal.pone.0012303 PMC2924888

[45] Courgnaud V, Seng R, Becquart P, Boulahtouf A, Rouzioux C, et al (2007) HIV-1 co-infection prevalence in two cohorts of early HIV-1 seroconverters in France. AIDS 21: 1055–1056. 17457105 10.1097/QAD.0b013e32810c8be1

[46] van der Kuyl A, Zorgdrager F, Jurriaans S, Back N, Prins J, et al (2009) Incidence of human immunodeficiency virus type 1 dual infections in Amsterdam, The Netherlands, during 2003–2007. Clin Infect Dis 48: 973–978. 19231977 10.1086/597356

[47] Smith D, Richman D, Little S (2005) HIV superinfection. J Infect Dis 192: 438–444. 15995957 10.1086/431682

[48] Redd A, Mullis C, Serwadda D, Kong X, Martens C, et al (2012) The rates of HIV superinfection and primary HIV incidence in a general population in Rakai, Uganda. J Infect Dis 206: 267–274. 22675216 10.1093/infdis/jis325 PMC3415936

[49] Shankarappa R, Margolick J, Gange S, Rodrigo A, Upchurch D, et al (1999) Consistent viral evolutionary changes associated with the progression of human immunodeficiency virus type 1 infection. J Virol 73: 10489–10502. 10559367 10.1128/jvi.73.12.10489-10502.1999 PMC113104

[50] Lemey P, Pond SLK, Drummond AJ, Pybus OG, Shapiro B, et al (2007) Synonymous substitution rates predict HIV disease progression as a result of underlying replication dynamics. PLoS Comput Biol 3: e29 doi:10.1371/journal.pcbi.0030029 17305421 10.1371/journal.pcbi.0030029 PMC1797821

[51] Poon A, McGovern R, Mo T, Knapp D, Brenner B, et al (2011) Dates of HIV infection can be estimated for seroprevalent patients by coalescent analysis of serial next-generation sequencing data. AIDS 25: 2019–2026. 21832936 10.1097/QAD.0b013e32834b643c

[52] Leventhal GE, Kouyos R, Stadler T, von Wyl V, Yerly S, et al (2012) Inferring epidemic contact structure from phylogenetic trees. PLoS Comput Biol 8: e1002413 doi:10.1371/journal.pcbi.1002413 22412361 10.1371/journal.pcbi.1002413 PMC3297558

[53] Frost SD, Volz EM (2013) Modelling tree shape and structure in viral phylodynamics. Philos Trans R Soc Lond B Biol Sci 368 20120208. 23382430 10.1098/rstb.2012.0208 PMC3678332

[54] Brown AJL, Lycett SJ, Weinert L, Hughes GJ, Fearnhill E, et al (2011) Transmission network parameters estimated from HIV sequences for a nationwide epidemic. J Infect Dis 204: 1463–1469. 21921202 10.1093/infdis/jir550 PMC3182313

