# Supplementary figures and images for: HIV-1 Transmission during Early Infection in Men Who Have Sex with Men: A Phylodynamic Analysis
Source: PLoS Med. 2013 Dec 10;10(12):e1001568. doi: 10.1371/journal.pmed.1001568 (PMC3858227; doi:10.1371/journal.pmed.1001568)

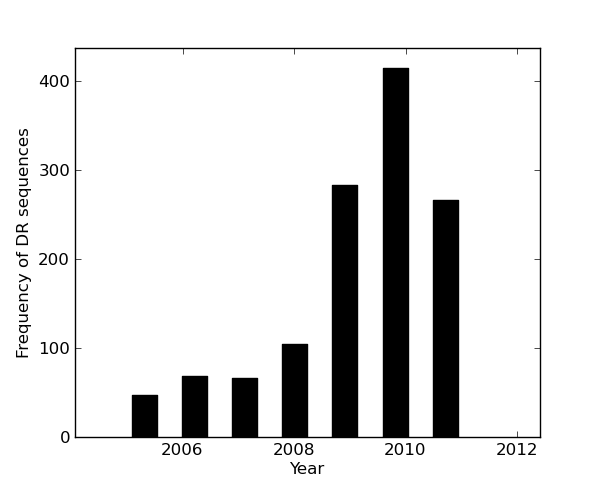

Supplement: Figure S1 — Number of HIV sequences sampled in Michigan by year. (PNG) [file pmed.1001568.s002.png]

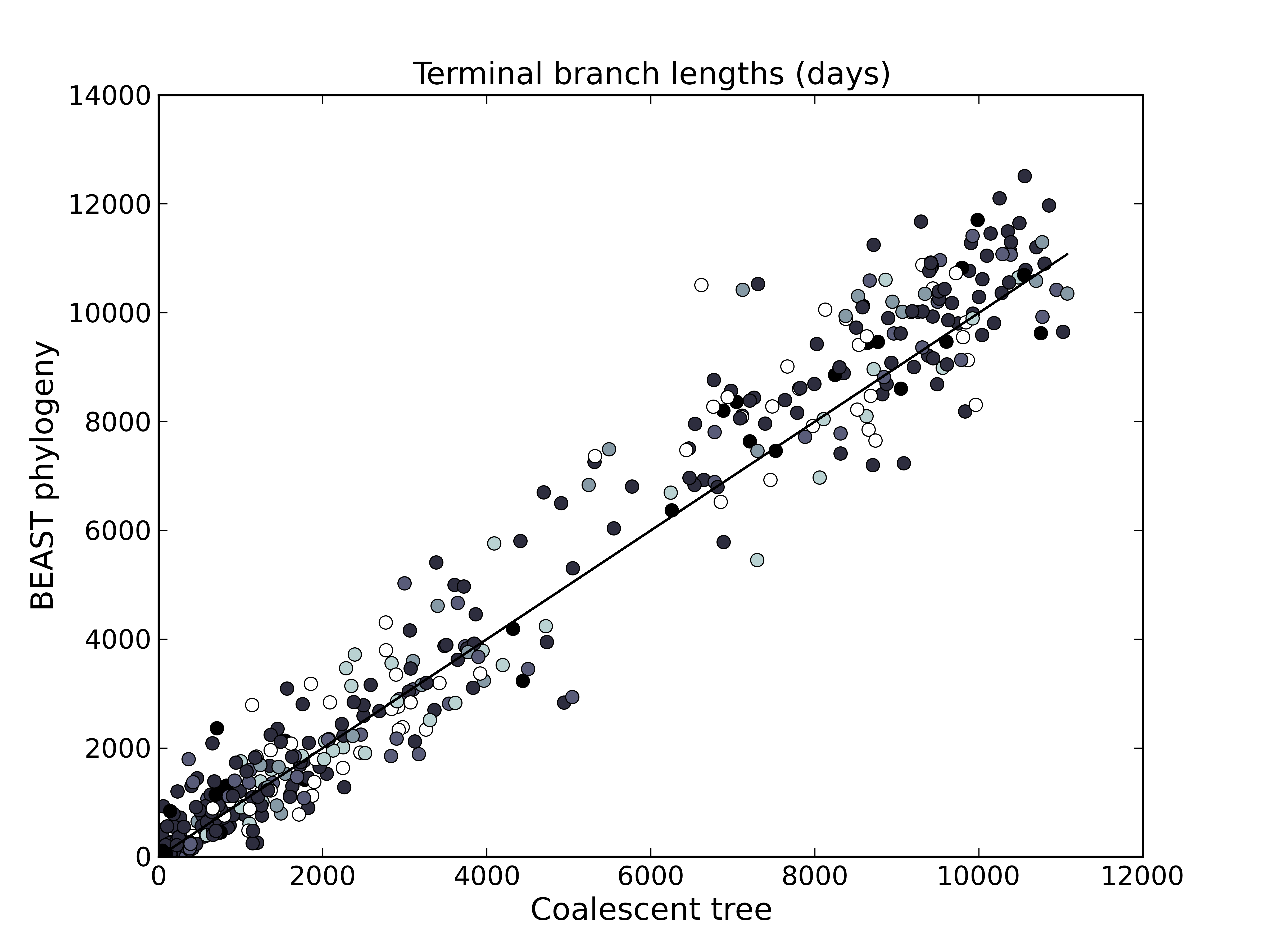

Supplement: Figure S2 — Comparison of estimated terminal branch lengths from relaxed clock phylogeny and the true branch lengths from a simulated tree. Color indicates the stage of infection of patient at time of sampling. Darker colors indicate patients sampled earlier in the infectious period. (PNG) [file pmed.1001568.s003.png]

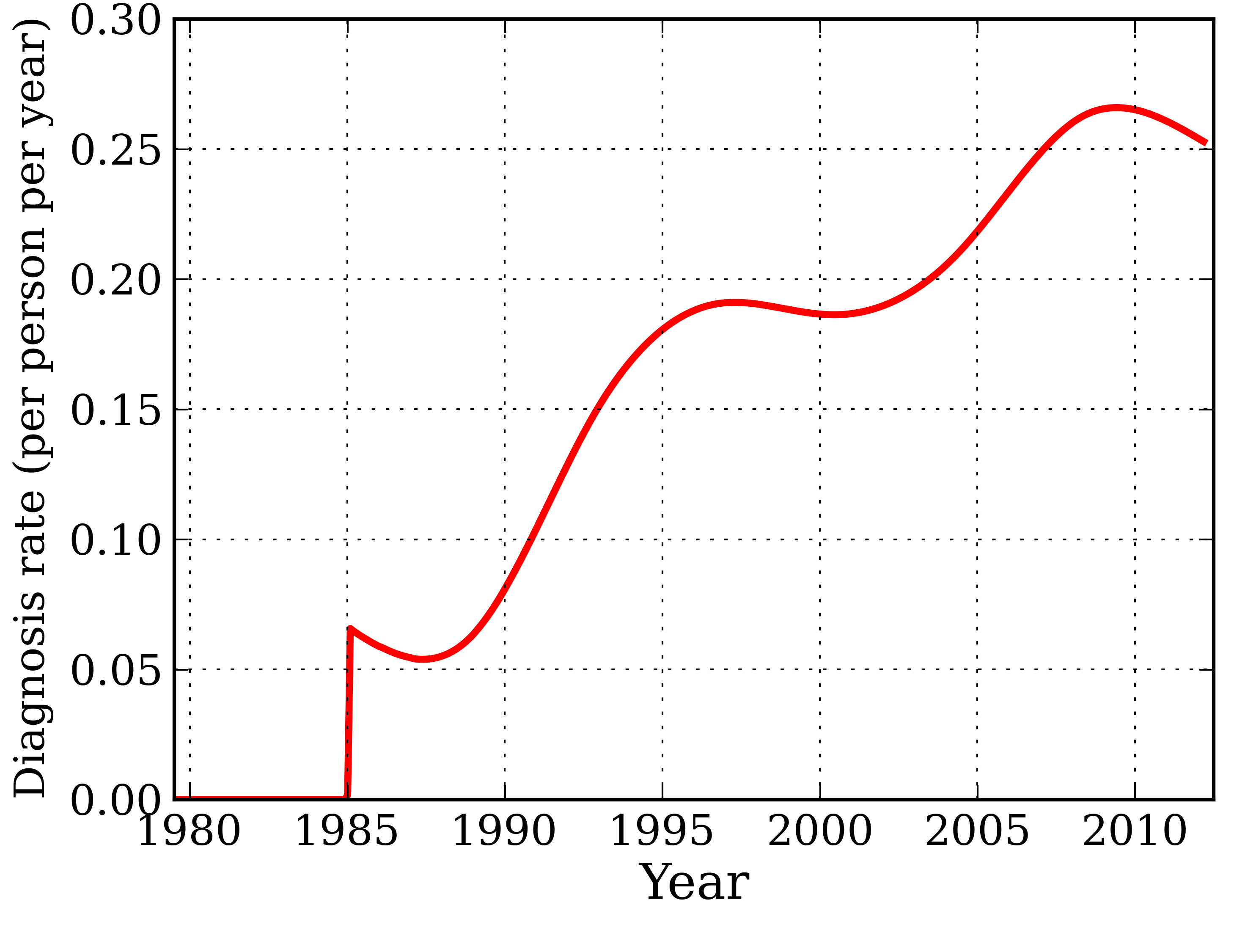

Supplement: Figure S3 — Estimated diagnosis rates over time. (TIFF) [file pmed.1001568.s004.tiff]

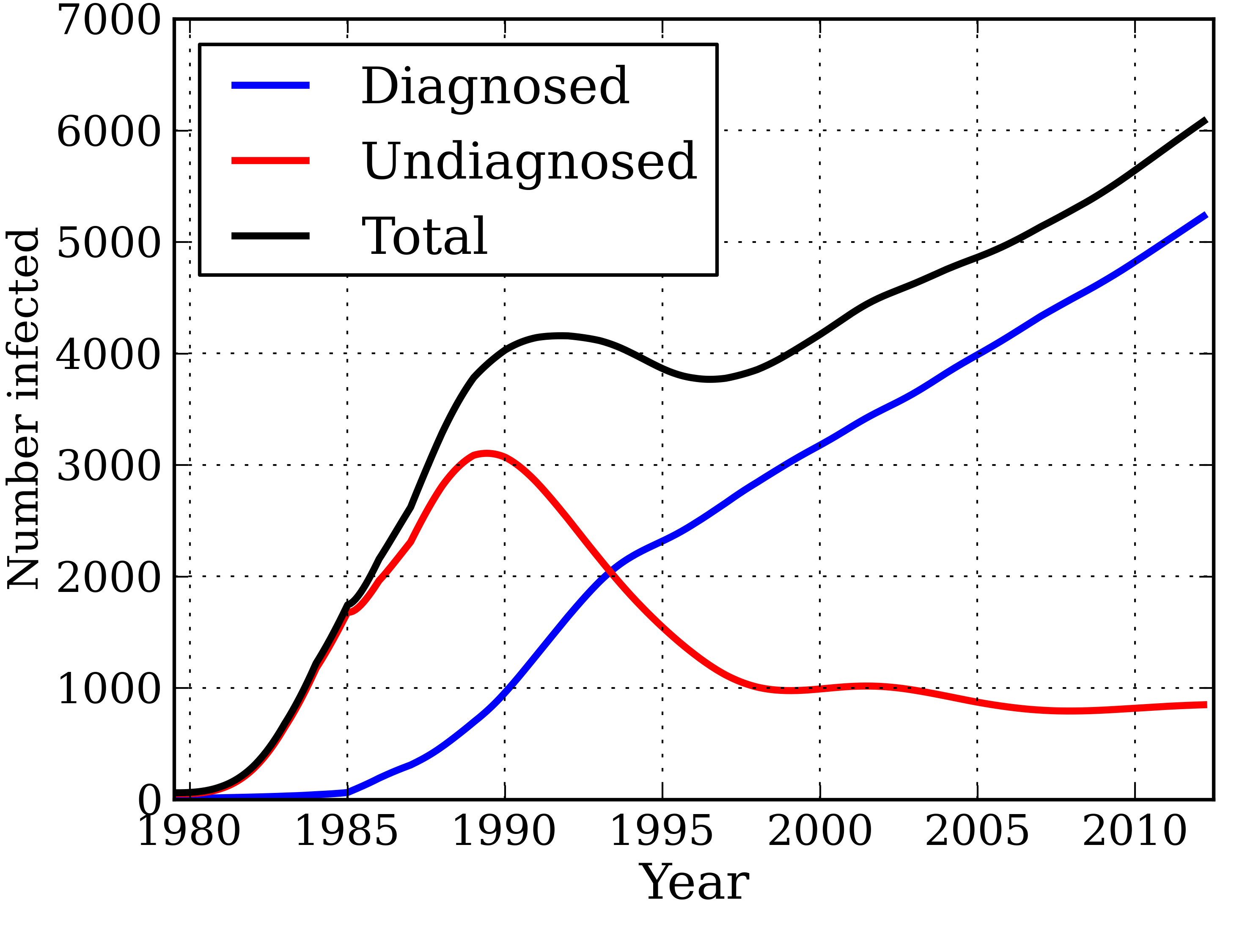

Supplement: Figure S4 — Estimated prevalence of infection over time. (TIFF) [file pmed.1001568.s005.tiff]

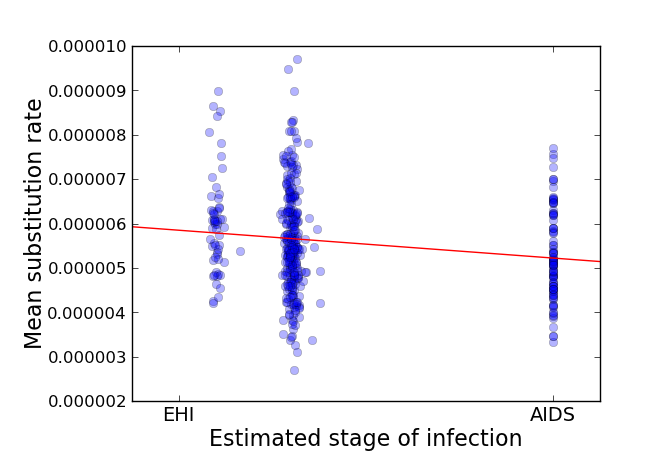

Supplement: Figure S5 — HIV nucleotide substitution rate and stage of infection. Blue points: the mean substitution rate is compared to the estimated stage of infection. The substitution rate for each patient corresponds to an external branch in the relaxed clock phylogeny estimated with BEAST. The stage of infection is estimated from AIDS-defining illness, the frequency of ambiguous sites of the HIV sequence, and CD4 as described in “Estimating Stage of Infection” in Text S4. Red line: linear regression. (PNG) [file pmed.1001568.s006.png]

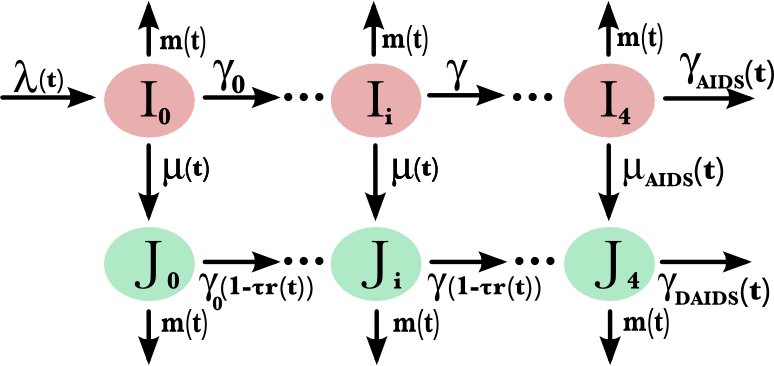

Supplement: Figure S6 — A flow diagram representing transitions made by infected individuals in the HIV model. Boxes represent categories of individuals who are infected with HIV and who may be diagnosed or undiagnosed in any of five stages of infection. Arrows represent the time-varying rates with which individuals transition between categories. (PNG) [file pmed.1001568.s007.png]

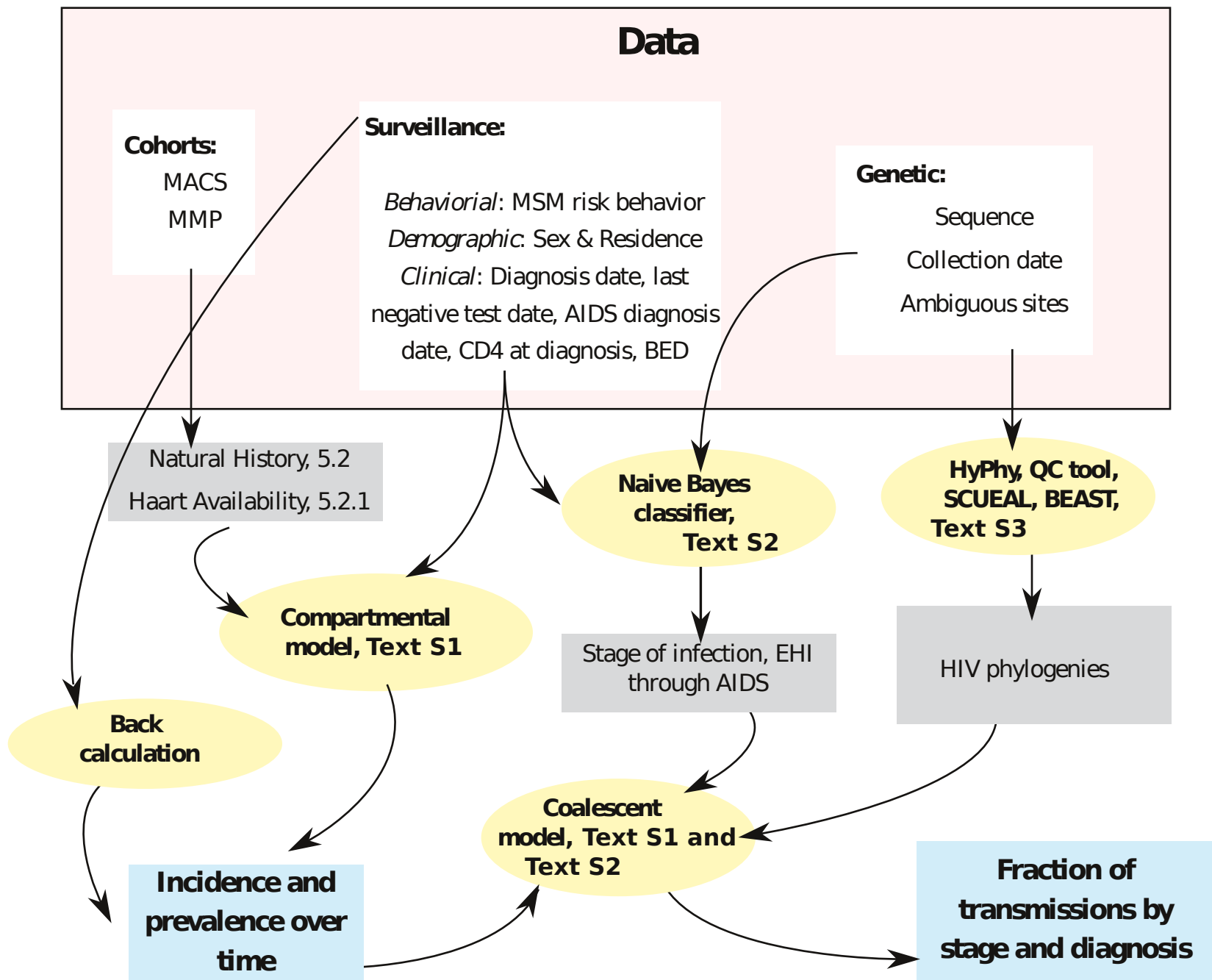

Supplement: Figure S7 — A schema illustrating how data were used at each stage of the analysis and how each analysis method was used to generate each result. The corresponding supporting text file that discusses each method is also listed. Primary data are shown in the red rectangle, procedures are shown in yellow ellipses, intermediate results are shown in grey rectangles, and final results are shown in blue rectangles. (PDF) [file pmed.1001568.s008.pdf]

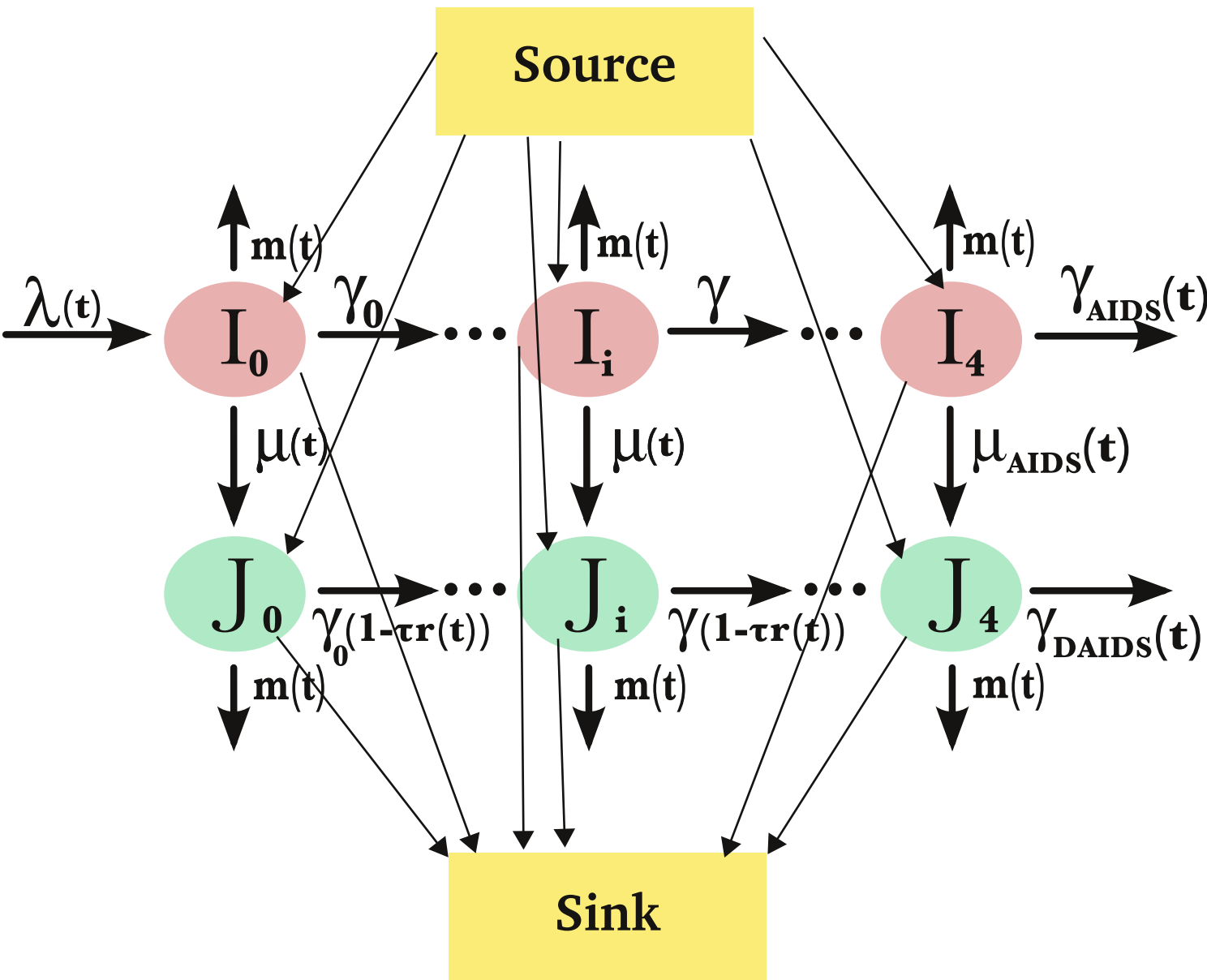

Supplement: Figure S8 — A flow diagram representing transitions made by infected individuals in the source–sink HIV model. Boxes represent categories of individuals who are infected with HIV and who may be diagnosed or undiagnosed in any of five stages of infection. Arrows represent the time-varying rates with which individuals transition between categories. The source and sink compartments represent migration of viral lineages in and out of the Detroit MSM risk group. (PDF) [file pmed.1001568.s009.pdf]

## Time from EHI to AIDS

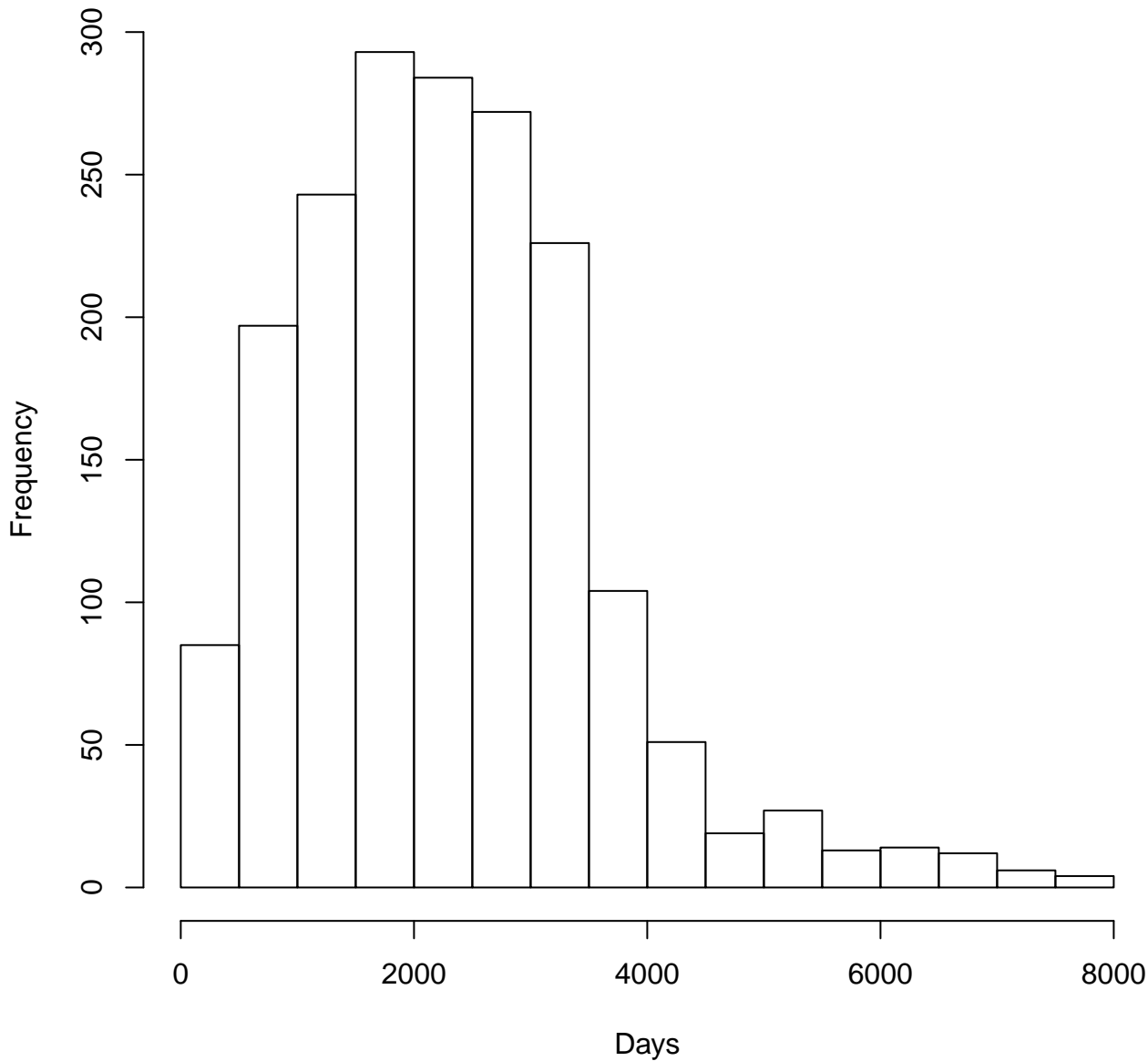

Supplement: Figure S9 — Simulated durations of chronic infection, which we define as the interval from the end of early HIV infection to the beginning of AIDS. Data are simulated based on data from the Multicenter AIDS Cohort Study. (PDF) [file pmed.1001568.s010.pdf]

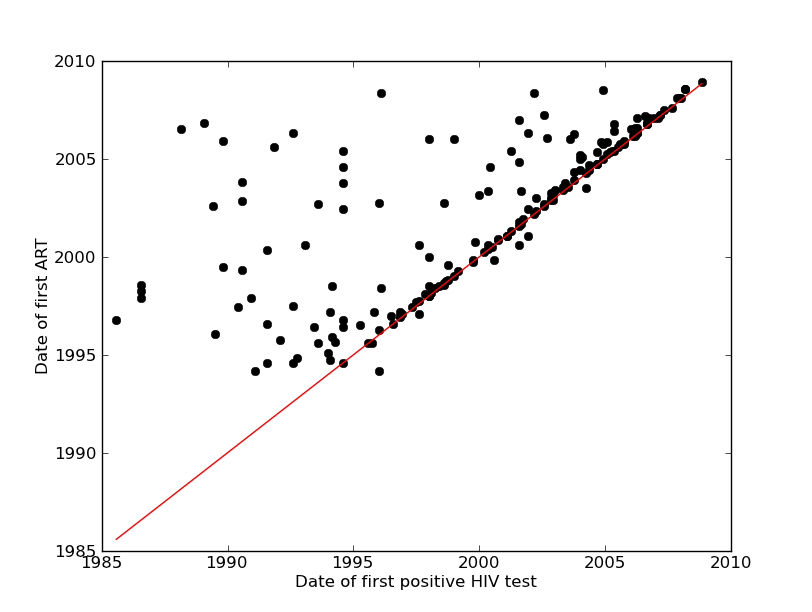

Supplement: Figure S10 — Antiretroviral uptake and usage through time. Left: Date of diagnosis and self-reported date of first antiretroviral therapy usage for participants in the Medical Monitoring Project in Michigan. Instances in which first antiretroviral therapy usage precedes diagnosis may be due to self-administered prophylaxis or due to reporting error. Right: The estimated HAART availability, as a function of time. (PNG) [file pmed.1001568.s011.png]

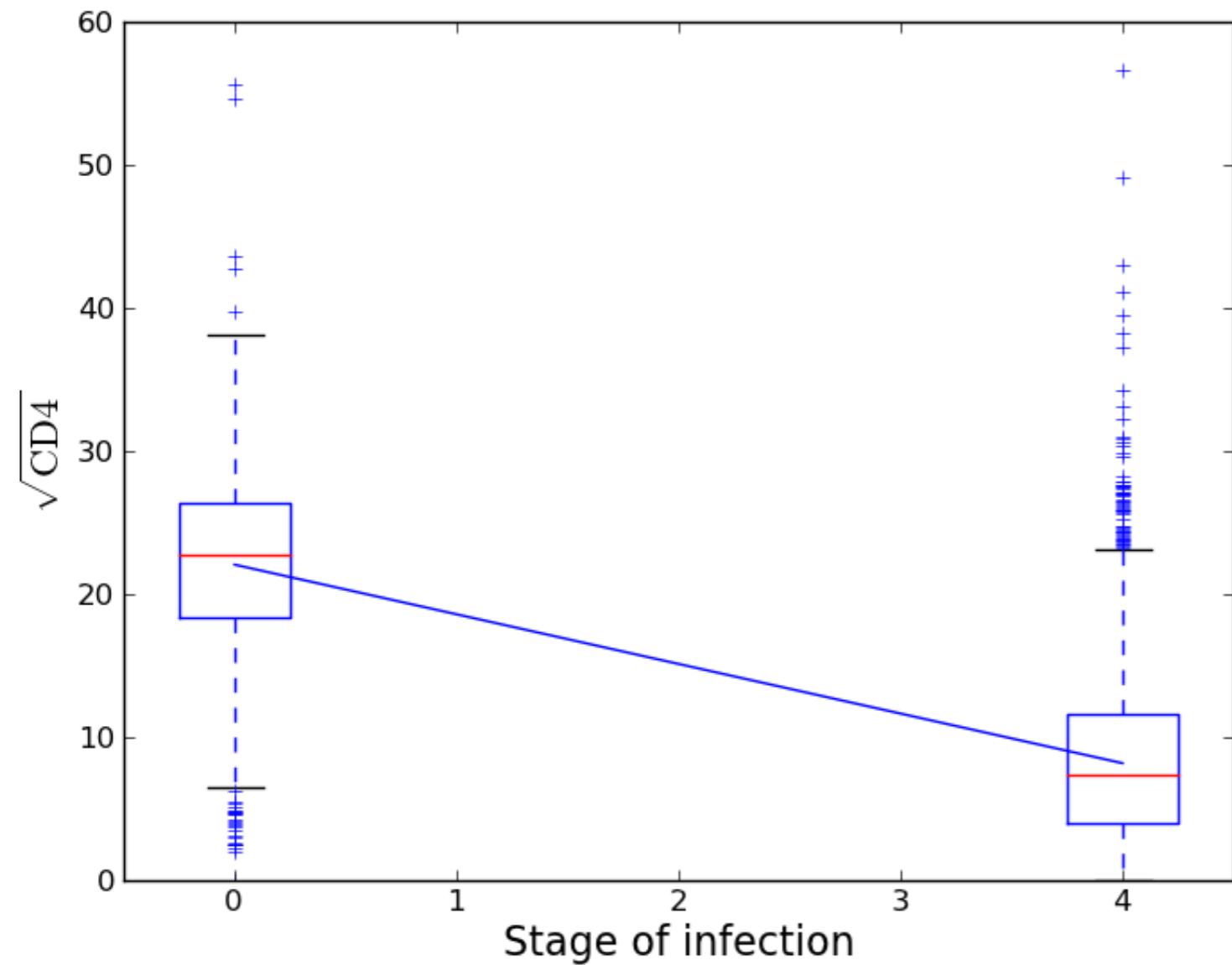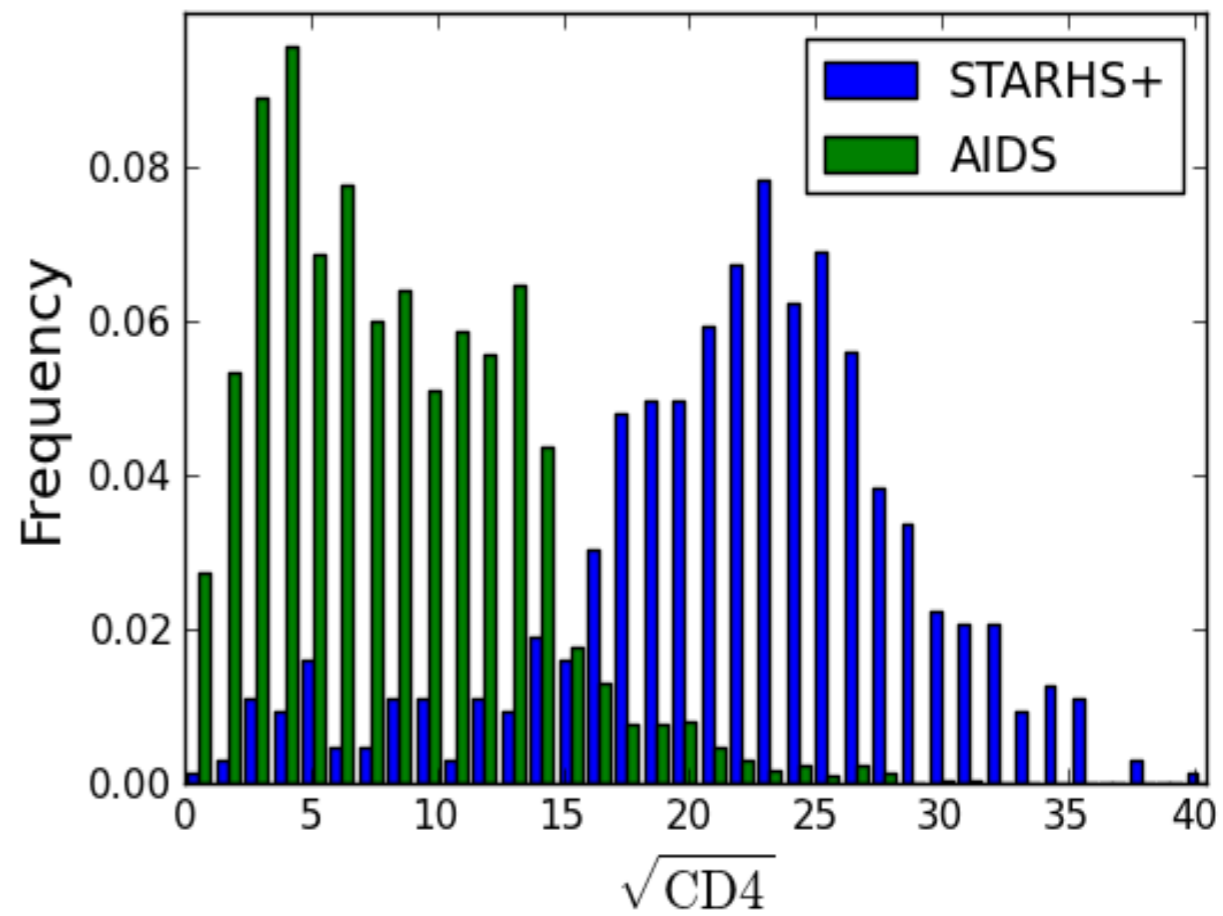

Supplement: Figure S11 — CD4 cell count by stage of infection at time of diagnosis. Left: A linear regression fit to the distribution of root CD4 counts for EHI and AIDS. Right: The distribution of root CD4 counts for EHI and AIDS. (PDF) [file pmed.1001568.s012.pdf]

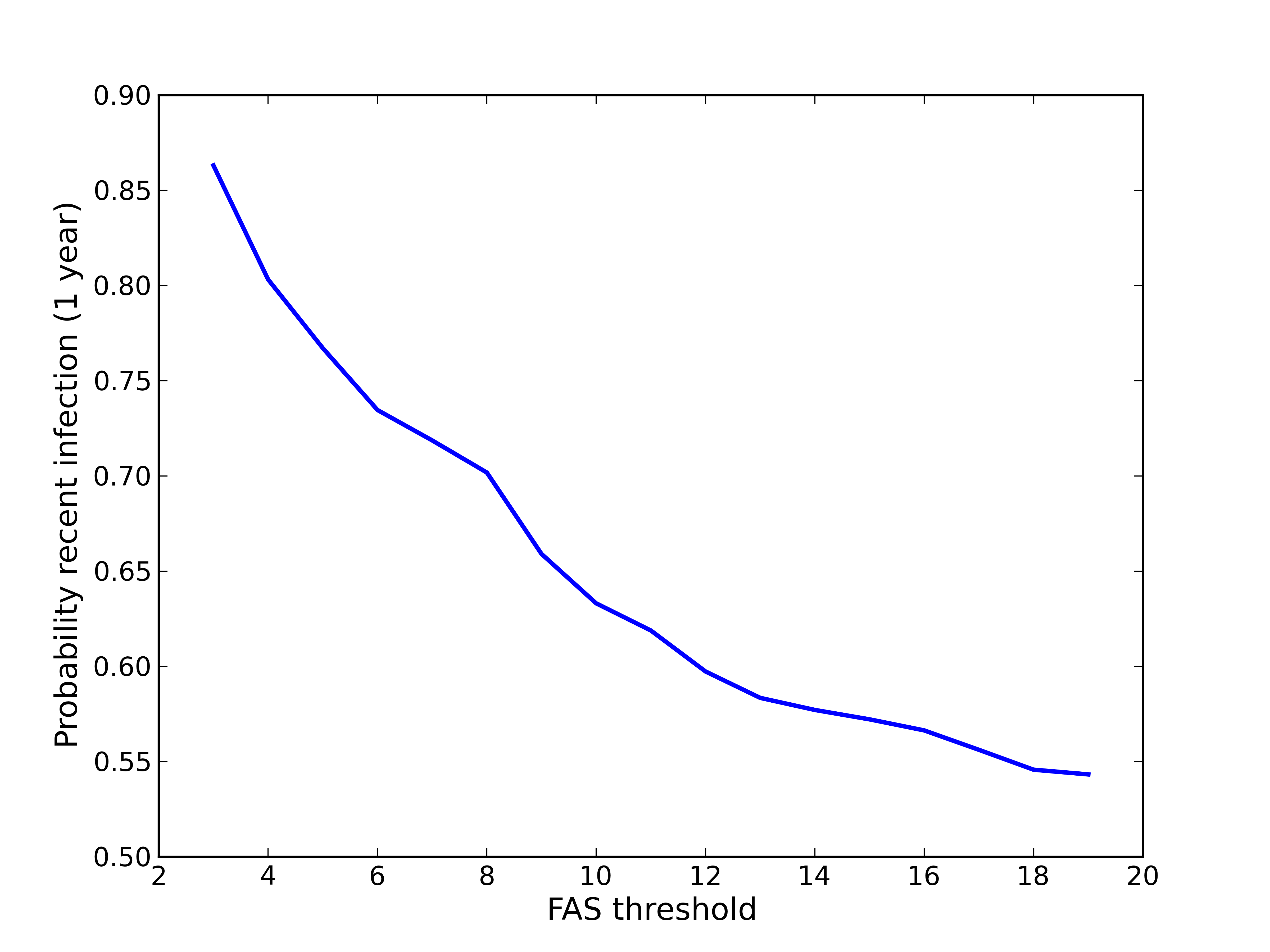

Supplement: Figure S12 — The probability that a sequence originated from a patient with early HIV infection if the number of ambiguous sites is less than the given threshold (positive predictive value). (PNG) [file pmed.1001568.s013.png]

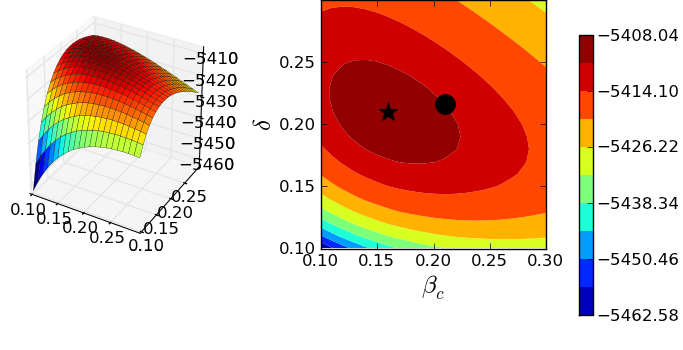

Supplement: Figure S13 — Likelihood surface for simulation experiment with demographic stochasticity. Left: The coalescent likelihood versus βc and δ. Right: Contour plot of the coalescent likelihood. The innermost contour shows all points within two log units of the maximum of the likelihood surface. The black circle indicates the true parameter value corresponding to the MLE in the main text. The black star indicates the maximum of the likelihood in the simulation experiment. (PNG) [file pmed.1001568.s014.png]

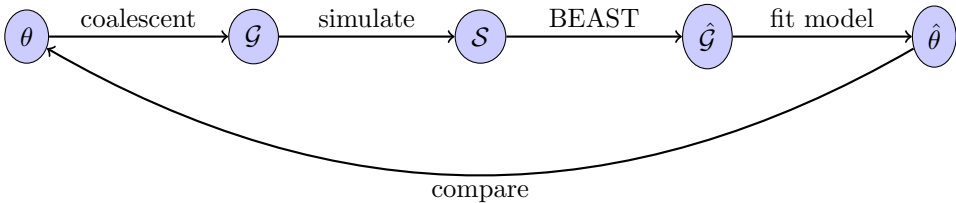

Supplement: Figure S14 — A flow-chart representation of the simulation experiment to determine the robustness of inferences to phylogenetic error. (PDF) [file pmed.1001568.s015.pdf]

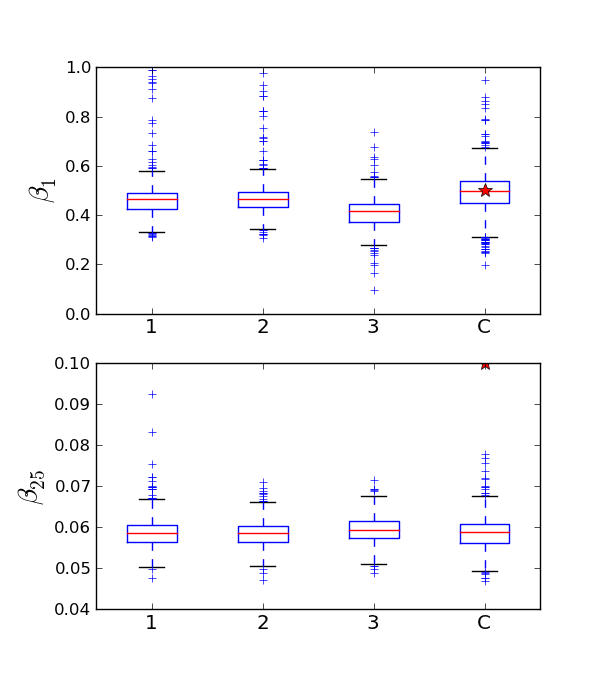

Supplement: Figure S15 — Estimated posteriors for the parameters β1 and β25 based on three trees estimated independently from BEAST. Also shown are estimates based on the true coalescent tree, and the parameters used to generate the coalescent tree (red star). (PNG) [file pmed.1001568.s016.png]

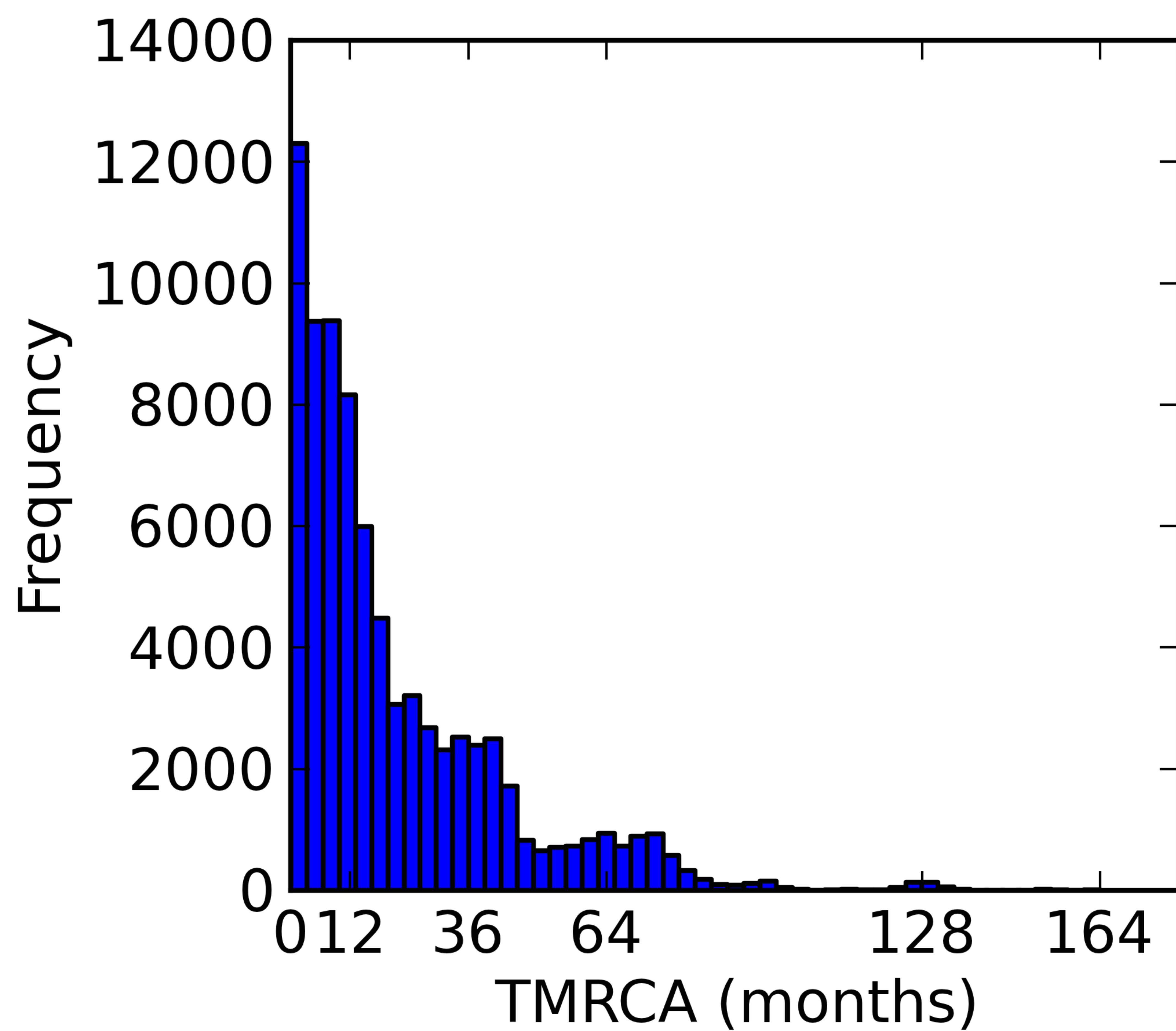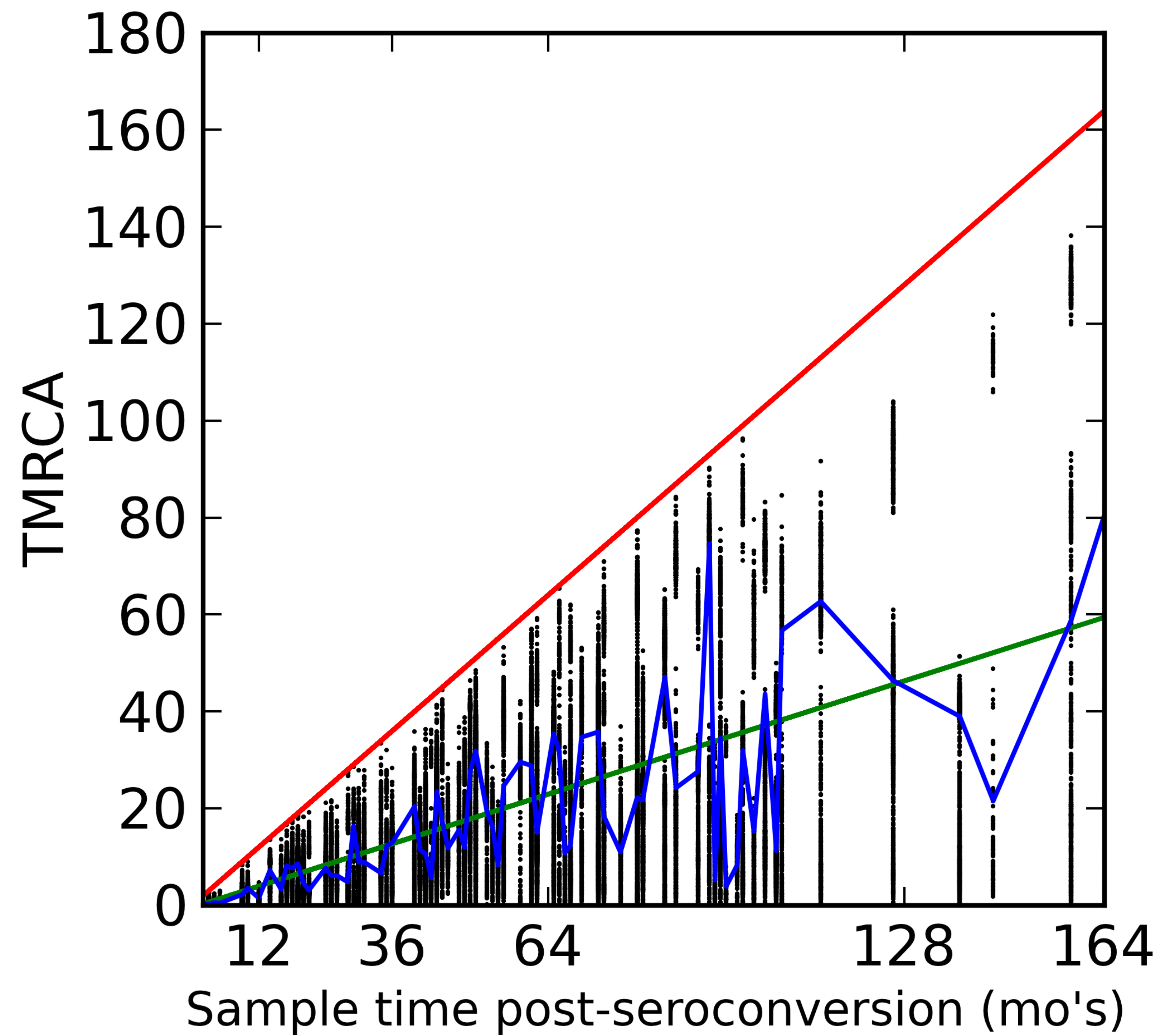

Supplement: Figure S16 — Within host coalescence of HIV lineages. Left: Histogram of intra-host coalescent times (TMRCA) for all pairs of isochronously sampled sequences in [49]. Right: Time to seroconversion versus intra-host coalescent times for all pairs of isochronously sampled sequences for nine patients in [49]. The blue line shows the median TMRCA at each sample point, and the green line shows a linear regression of TMRCA on time since seroconversion. (PDF) [file pmed.1001568.s017.pdf]

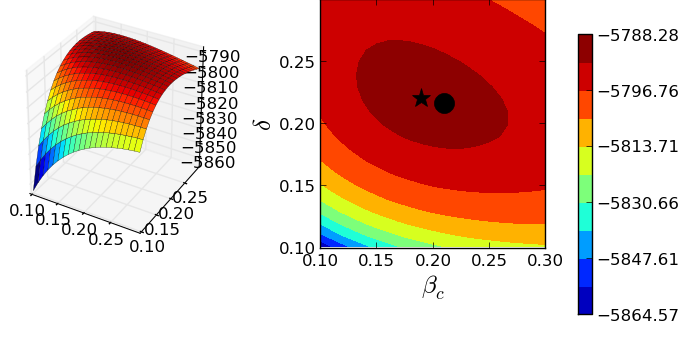

Supplement: Figure S17 — Likelihood surface for simulation experiment with intra-host evolution. Left: The coalescent likelihood versus βc and δ for a simulated tree where nodes correspond to intra-host coalescent events rather than transmission events. Right: Contour plot of the coalescent likelihood. The innermost contour shows all points within two log units of the maximum of the likelihood surface. The black circle indicates the true parameter value corresponding to the MLE in the main text. The black star indicates the maximum of the likelihood in the simulation experiment. (PNG) [file pmed.1001568.s018.png]

A

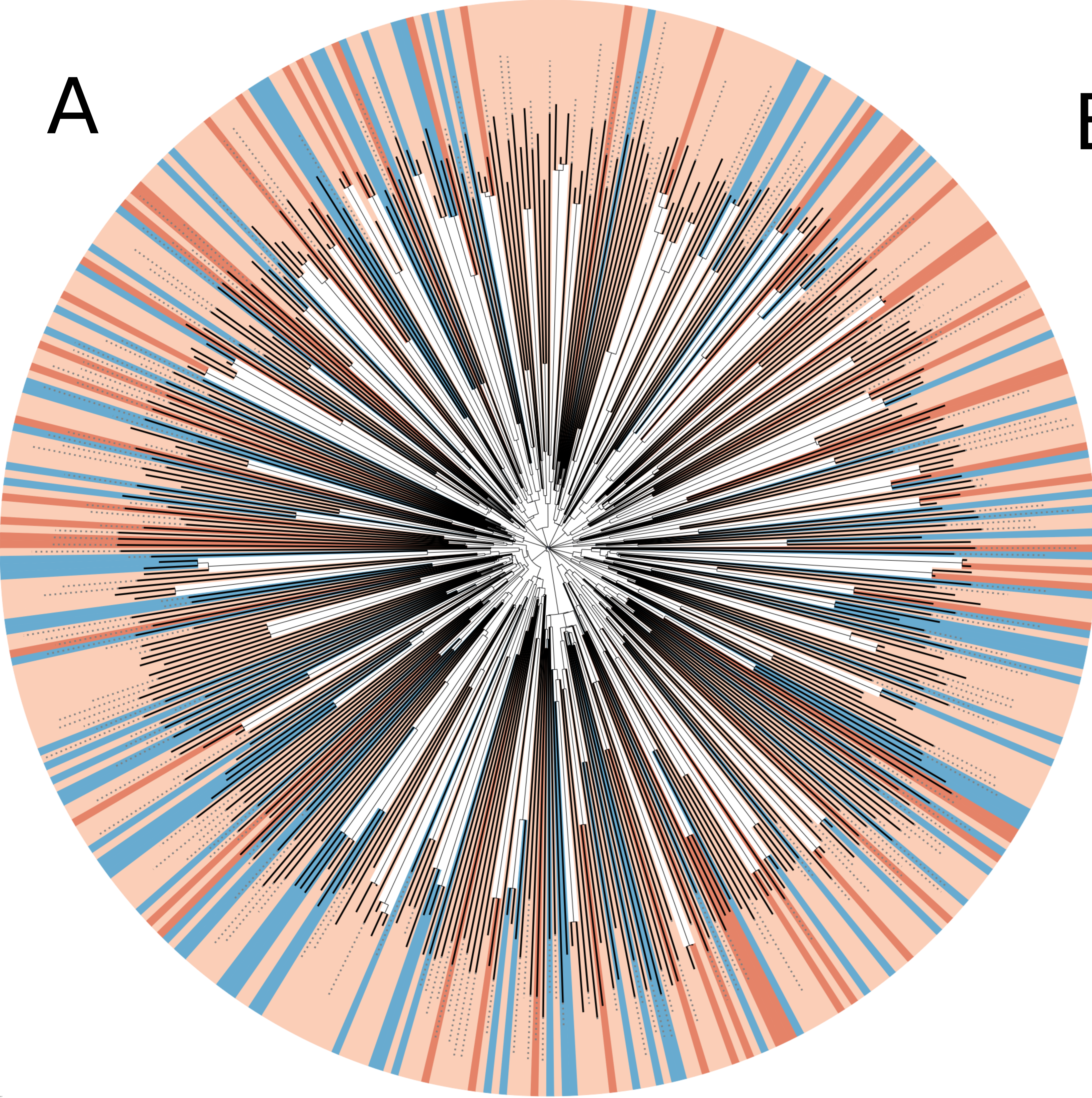

B

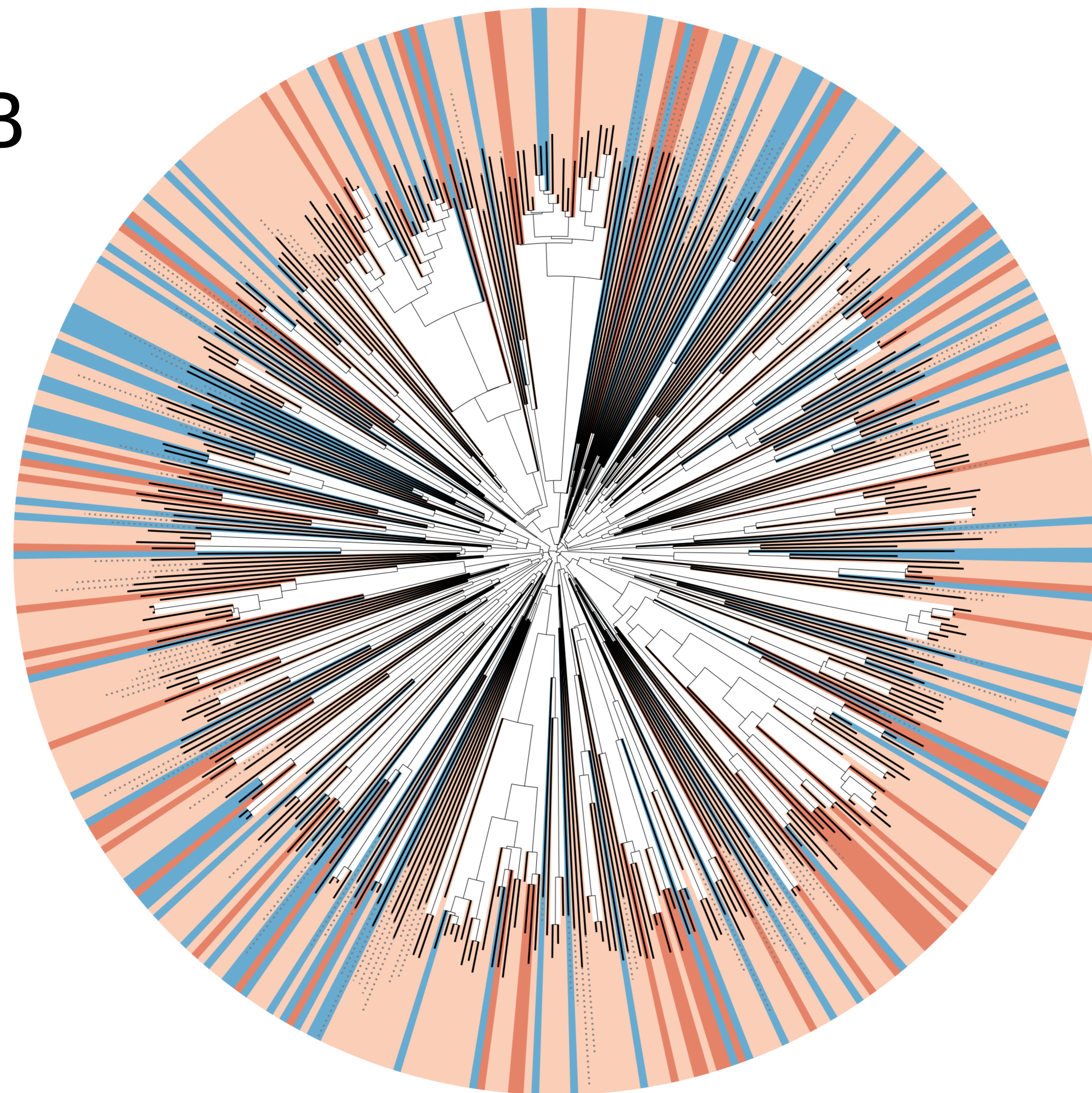

C

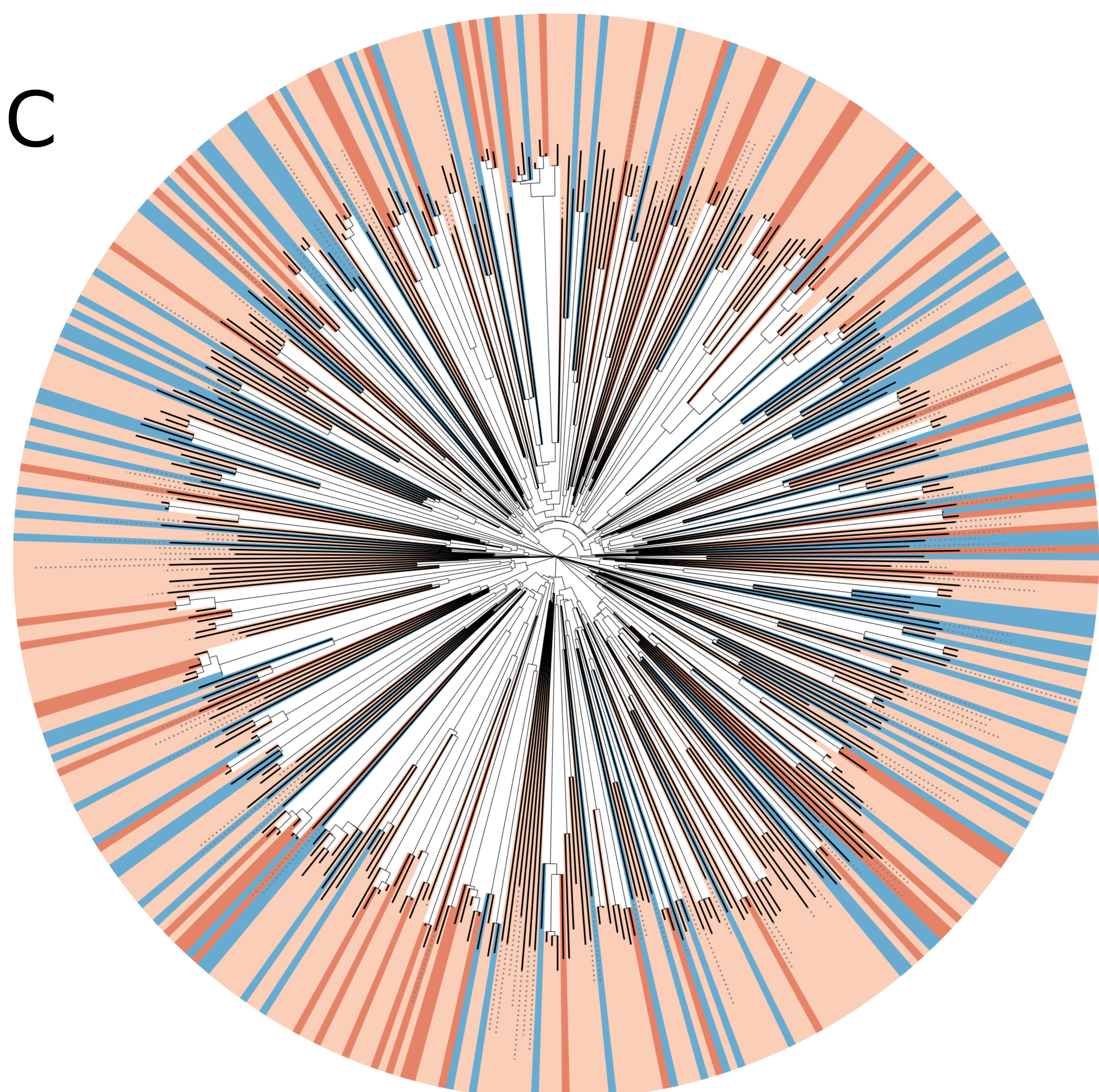

Supplement: Figure S18 — Simulated coalescent trees for HIV models. (A) A simulated coalescent tree under a scenario where individuals with EHI transmit at a rate equal to that of individuals with chronic infection or AIDS. (B) The HIV-1 phylogeny of 437 patients. (C) A simulated coalescent tree such that individuals with EHI transmit at a greater rate than those with chronic infection, as described by the MLE model fit in the main text. Terminals of the tree are colored according to stage of infection of the patient at the time of sampling. Red indicates those sampled during EHI or chronic infection stages. Blue indicates sampling during AIDS. (PDF) [file pmed.1001568.s019.pdf]
