# Supplementary material for: HIV-1 Transmission during Early Infection in Men Who Have Sex with Men: A Phylodynamic Analysis
Source: PLoS Med. 2013 Dec 10;10(12):e1001568. doi: 10.1371/journal.pmed.1001568 (PMC3858227; doi:10.1371/journal.pmed.1001568)
Supplement: Table S1 — Model comparisons. (PDF) [file pmed.1001568.s020.pdf]

Free parameters	EHI TF	Diagnosed TF	$\Delta ln(\mathcal{L})$	AIC
β_c, δ, β_a	0.431024	0.508944	0	24448.896273
β_c, β_a	0.430984	0.508803	-0.004635	24446.905544
β_c, δ	0.500422	0.531187	-28.160443	24503.21716
β_c	0.500496	0.531007	-28.162529	24501.221332
δ	0.17666	0.293641	-103.756317	24652.408908
