# Supplementary material for: HIV-1 Transmission during Early Infection in Men Who Have Sex with Men: A Phylodynamic Analysis
Source: PLoS Med. 2013 Dec 10;10(12):e1001568. doi: 10.1371/journal.pmed.1001568 (PMC3858227; doi:10.1371/journal.pmed.1001568)
Supplement: Text S1 — Detailed methods for phylogenetic analysis. (PDF) [file pmed.1001568.s021.pdf]

Supporting methods for *HIV-1 Transmission During Early Infection in Men Who Have Sex with Men: A Phylodynamic Analysis*

Erik M Volz*[1], Edward Ionides[2], Ethan Romero Severson[3],
Mary Grace Brandt[4], Eve Mokotoff[4], James S Koopman[5]

* Corresponding author: e.volz@imperial.ac.uk

1. Department of Infectious Disease Epidemiology, Imperial College London, UK
2. Department of Statistics, University of Michigan- Ann Arbor, USA
3. Theoretical Biology and Biophysics, Los Alamos National Laboratory, Los Alamos,New Mexico
4. Michigan Department of Community Health, Detroit, MI, USA
5. Department of Epidemiology, University of Michigan- Ann Arbor, USA

September 27, 2013

S1 Phylogenetic analysis

We analyzed of 2808 HIV-1 partial-*pol* sequences collected from MSM in Southeastern Michigan between 2004 and April 2012 inclusively. All sequences consisted of genes encoding Protease and Reverse Transcriptase and were originally collected for the purpose

of drug resistance testing. Clinical covariates of patients were associated with each sequence, including CD4 cell count, viral load, BED test result, presence of AIDS defining illnesses, and date of last negative HIV test. Demographic covariates were also available, including age at diagnosis, race, residence (county), and primary risk behavior associated with HIV transmission. We considered only subtype-B sequences from men residing in the Detroit metropolitan area (DMA) whose primary risk behavior was sex with men.

When more than one sequence was available for a patient, we excluded all but the one collected nearest to the diagnosis date.

Sequences with fewer than 1150 non-missing characters were excluded. Subtype was determined using Hyphy [1], and only sequences identified as subtype B were retained. Hyphy was also used to detect possible intra-subtype recombinants, which were excluded from subsequent analysis. Reverse transcriptase and protease sequences were aligned separately against a subtype B reference (HXB2) and reassembled using Biopython [2]. A list of sites associated with drug resistance was collected from the Stanford HIV database [3]. Because these sites are under selection, these sites were masked (treated as missing data) using Biopython. Subsequent phylogenetic and coalescent analysis was conducted on a subset of 662 sequences for which the sequence was collected within 12 months of diagnosis.

Each of 662 sequences was analyzed with BLAST to find a closest match in the LANL HIV database. This yeilded 100 unique matches. We realigned our data with the matches and included these in subsequent phylogenetic and coalescent analysis. In the coalescent analysis, these sequences were indicated as belonging to the 'source' population (see Text S1)

To speed phylogenetic estimation, the sequence alignment with $n = 662 + 100$ was divided into 9 parts by the following algorithm:

- A NJ tree was calculated for all sequences using the ape library in R and usign the TN93 with Γ rate variation.
- The tree was re-rooted with path-o-gen [4] and using known sample dates.
- Recursively select internal branches with 50-150 descendants. Prune this clade from the tree, and generate new sequence alignment corresponding to its terminals. Repeat until no terminals are left in the tree.

This algorithm was implemented in Biopython and ETE [5].

Phylogenies were estimated using BEAST 1.6.3 [6] which is a Bayesian relaxed clock method that provides an estimate of branch lengths and node heights in scaled in calendar time. We used a relaxed molecular clock [7] with discretized Gamma rate variation between sites (4 categories). The model of nucleotide substitution was GTR with a fraction I invariant sites. The starting tree was found by UPGMA. The evolutionary model assumed a skyride [8] coalescent prior of demographic history. The MCMC ran for 250 million iterations and sampling at every 5×10^4 iterations. Results were analyzed in Tracer [6], and each log file was checked for consistency with results from other replicates. Combined log files had and effective sample size (ESS) of > 500 for all parameters.

The coalescent analysis was performed on a tree derived from all 9 BEAST analyses:

- A tree is sampled from each of 9 posterior distributions.
- To render branch lengths comparable between trees, the substitution rates are adjusted and branch lengths changed accordingly.
  - The mean substitution rate across all trees is estimated as

$$\hat{s} = \sum_k \sum_i l_{ki} s_{ki} / \left(\sum_k \sum_i l_{ki} \right),$$

where l_{ki} is the length of branch i on tree k, s_{ki} is the substitution rate on branch i of tree k.

– The mean substitution rate for each of 9 trees was also calculated:

$$\hat{s}_k = \sum_i l_{ki} s_{ki} / \left(\sum_i l_{ki} \right).$$

– The height of each tree k and all nodes were rescaled by a factor $\hat{s}_k/\hat{s}$. Branch lengths are recomputed given new node heights.

– If negative branch lengths are introduced by rescaling, than the original branch length is restored.

- The trees are joined at a polytomous root with a height equal to that of the highest node among the 9 trees.

For subsequent model fitting using coalescent methods, we used a sample of 10 trees from each of 9 posterior distributions corresponding to each BEAST analysis. Coalescent models were fitted by averaging the likelihood from 10 trees (see Text S2).

Supplementary References

1. Pond S, Muse S (2005) Hyphy: hypothesis testing using phylogenies. Statistical Methods in Molecular Evolution : 125–181.

2. Cock PJ, Antao T, Chang JT, Chapman BA, Cox CJ, et al. (2009) Biopython: freely available python tools for computational molecular biology and bioinformatics. Bioinformatics 25: 1422–1423.

3. Bennett D, Camacho R, Otelea D, Kuritzkes D, Fleury H, et al. (2009) Drug resistance mutations for surveillance of transmitted HIV-1 drug-resistance: 2009 update. PLoS One 4: e4724.

4. Rambaut A (2011). Path-O-Gen: A tool for investigating the temporal signal and 'clocklikeness' of molecular phylogenies. http://tree.bio.ed.ac.uk/software/pathogen/.

5. Huerta-Cepas J, Dopazo J, Gabaldón T (2010) Ete: a python environment for tree exploration. BMC bioinformatics 11: 24.

6. Drummond A, Rambaut A (2007) BEAST: Bayesian evolutionary analysis by sampling trees. BMC Evol Biol 7: 214.

7. Drummond A, Ho S, Phillips M, Rambaut A (2006) Relaxed phylogenetics and dating with confidence. PLoS Biol 4: e88.

8. Minin V, Bloomquist E, Suchard M (2008) Smooth skyride through a rough skyline: Bayesian coalescent-based inference of population dynamics. Mol Biol Evol 25: 1459–1471.
