# Supplementary material for: HIV-1 Transmission during Early Infection in Men Who Have Sex with Men: A Phylodynamic Analysis
Source: PLoS Med. 2013 Dec 10;10(12):e1001568. doi: 10.1371/journal.pmed.1001568 (PMC3858227; doi:10.1371/journal.pmed.1001568)
Supplement: Text S2 — Detailed description of HIV transmission model. (PDF) [file pmed.1001568.s022.pdf]

Supporting methods for *HIV-1 Transmission During Early Infection in Men Who Have Sex with Men: A Phylodynamic Analysis*

Erik M Volz*[1], Edward Ionides[2], Ethan Romero Severson[3], Mary Grace Brandt[4], Eve Mokotoff[4], James S Koopman[5]

* Corresponding author: e.volz@imperial.ac.uk

1. Department of Infectious Disease Epidemiology, Imperial College London, UK

2. Department of Statistics, University of Michigan- Ann Arbor, USA

3. Theoretical Biology and Biophysics, Los Alamos National Laboratory, Los Alamos,New Mexico

4. Michigan Department of Community Health, Detroit, MI, USA

5. Department of Epidemiology, University of Michigan- Ann Arbor, USA

September 27, 2013

S1 Text S1: Supporting methods and simulation results

The results presented in the main text involved a complex synthesis of different data sets and different methods. Figure S7 illustrates how each dataset was used and how each analysis method was used to generate each result. The figure also indicates which section in the supplement contains additional information about each step of the analysis. The main results are

1. Estimated incidence and prevalence over times
2. Fraction of transmissions attributable to individuals at different stages of infection and those who are diagnosed/undiagnosed

There are a variety of ways to estimate incidence and prevalence, and we have presented two. All of these make use of surveillance data and data that is informative about the stage of infection at diagnosis. The genetic data is not essential to estimating incidence and prevalence. In contrast, the genetic data is central to estimating the source of transmissions, and a more complex analytical pipeline is required to make use of the genetic data, involving sequence alignment, quality control, phylogenetic estimation and finally, the fitting of a coalescent model.

S1 Epidemiological model

Estimation of HIV incidence is complicated by the long infectious period and long delay between infection and diagnosis. Diagnosis rates have changed substantially over the course of the epidemic and may also vary between risk-groups and over the course of

infection. When estimating HIV incidence, an inherent tradeoff must be recognized between time-dependent diagnosis rates and the latent unobserved incidence responsible for generating observed diagnoses. Does an increase in the number of diagnosed cases reflect increasing incidence or greater testing and diagnosis rates? One approach to disentangling these effects is based on the presence or absence of various antigens and antibodies that occur at different times after infection [1,2]. However, these assays are often problematic. False positive can result for patients diagnosed before or concurrently with AIDS, which has limited the generalizeability to settings with different rates of AIDS diagnosis [3]. Current antibody assays are limited to a detection window of 162 days post-infection [4], which also limit the resolution of these tests for determining recent infections.

In locales which lack a centralized HIV surveillance system, transmission system models, usually formulated with ordinary differential equations (ODEs), are fitted to seroprevalence survey data. There are relatively few transmission models which attempt to integrate clinical data regarding stage of infection for estimation of HIV incidence. We follow the approach taken in Bezemer et al. [5], which integrates clinical data where it is available with an explicit HIV transmission model. This model describes incidence and prevalence over time using a system of ordinary differential equations, but also describes the natural history of infection as progressing through EHI, 3 chronic stages, and AIDS, which closely reproduces empirical observations regarding the time from infection to AIDS (section S2). This model tracks both the number of undiagnosed and diagnosed infected individuals over time. Diagnosis rates vary over time, and also between stages of infection. Fitting of the model can therefore make use of clinical variables which are informative of the stage of infection at the time of diagnosis. This model avoids mechanistic assumptions about how incidence depends on the number or frequency of susceptible individuals. Similar to models proposed by Hogan et al. [6], incidence and diagnosis rates are described by

smoothed cubic B-splines. This model is based on implicit conditions that risk behavior and sexual network patterns are homogeneous within each stage of infection. The model is illustrated in figure S6.

The HIV model was derived from two recently published models described below. In [6], smoothing splines were used to model incidence and diagnosis rates as a function of time. This has the advantage of not requiring assumptions about unknown mechanisms regarding contact rates, sexual networks and the number of susceptibles at risk of infection. This model, however, did not realistically model the natural history of infection or account for the effects of treatment or differential diagnosis rates. A similar model was developed in [5], which additionally used a compartmental structure to realistically model the natural history of infection and the effects of HAART.

The final model, elaborated below, consists of two smoothing splines for incidence and diagnosis rates, a compartmental model for the natural history of infection, parameters describing the initial conditions, and several parameters controlling the relative contribution of different stages of infection and diagnosed individuals to total transmissions. In contrast to most compartmental models, this eschews mechanistic descriptions of the way incidence scales with the numbers of susceptible individuals. The model does not contain terms describing the numbers of susceptibles, and does not have parameters describing contact rates, transmission probabilities, or sexual network structure. The model was selected for the estimation of incidence, diagnosis rates, and fraction of transmissions attributable to early infection.

Incidence of infection $\lambda(t)$ is modeled with a 3rd degree smoothing spline [7]. This has previously [6] been suggested as an appropriate model for HIV epidemics. The smoothing parameter for the spline was fixed at 0.2. Various number of control points between 7-36 for the spline function were tried and log likelihoods were compared after Nelder-Mead

optimization from 6000 random starting conditions. All results presented in the main text were based on a spline with 13 evenly spaced control points between 1975 and 2011 which was found to give good results. Splines were implemented using the *smooth.spline* function in R 2.15.1.

Following the model developed in [5], chronic HIV infection progresses through several stages of equal average duration. We find that three stages of chronic infection acurately reproduces the duration of chronic infection (see section S2). The rate of progression from stage i to $i+1$ is $\gamma = 1/2.1$ years^{-1}. This model yields a Gamma-distributed interval from the duration of chronic infection. Additionally, we include an early HIV infection (EHI) period before the first stage, which lasts 1 year on average ($\gamma_0 = 1/365$ day^{-1}). The EHI stage will sometimes be referred to as stage zero, and variables relating to this stage will appear with subscript 0. The final stage of infection is AIDS, which will be synonymous with stage 4.

Infected individuals are diagnosed at a rate $\mu(t)$ which varies over time. This rate is modeled with a 3rd degree smoothing spline with eight evenly spaced control points. $\mu(t)$ is the same for stages 0 through 3. The AIDS diagnosis rate $\mu_{AIDS}(t)$ is a piecewise-constant function chosen to reproduce the observed timeseries of HIV/AIDS diagnoses. Since the diagnosis rates change over time, the time interval from infection to diagnosis does not have a simple form.

I_0 and J_0 will respectively denote the number of undiagnosed and diagnosed infected individuals with EHI. I_i and J_i will respectively denote the number of undiagnosed and diagnosed infected individuals in stages $i = 1$ through $i = 4$ (AIDS).

The surveillance data was very complete with respect to deaths in diagnosed individuals and surveillance data also indicate if the deceased had been diagnosed with AIDS prior to death. The health department does annual death matching with the state vital

records department and periodic matching with the 'Social Security Death Master File' and the 'National Death Index' to obtain deaths on persons who have moved out of the state after diagnosis. We calculated empirical death rates for each year of surveillance data using the available death dates and the known number of diagnosed AIDS cases.

We assume that natural mortality (non-AIDS related) in undiagnosed individuals without AIDS is the same as for diagnosed individuals without AIDS. Natural mortality in infected individuals, that is, deaths not associated with HIV/AIDS, occurs at a rate $m(t)$. Mortality from undiagnosed AIDS cases occurs at the rate $\gamma_{AIDS}(t)$, and mortality from diagnosed AIDS cases occurs at the rate $\gamma_{DAIDS}(t)$. All of these mortality rates are piecewise constant functions with respect to time and are parameterized from surveillance data to reproduce the observed timeseries of non-AIDS and AIDS deaths. Mortality data was incomplete for 2009-11, so we used the estimated natural mortality rate in 2008 for those years.

The model for prevalence of infection over time comprises a system of 10 ODEs which

describe the time course for the I and J state variables:

$$
\begin{aligned}
\frac{\mathrm{d}}{\mathrm{d}t}I_0 &= \lambda(t) - (\gamma_0 + \mu(t) + m(t))I_0, \\
\frac{\mathrm{d}}{\mathrm{d}t}J_0 &= \mu(t)I_0 - (\gamma_0(1-\tau r(t)) + m(t))J_0, \\
\frac{\mathrm{d}}{\mathrm{d}t}I_1 &= \gamma_0 I_0 - (\gamma + \mu(t) + m(t))I_1, \\
\frac{\mathrm{d}}{\mathrm{d}t}J_1 &= \gamma_0 J_0 + \mu(t)I_1 - (\gamma(1-\tau r(t)) + m(t))J_1, \\
\frac{\mathrm{d}}{\mathrm{d}t}I_i &= \gamma I_{i-1} - (\gamma + \mu(t) + m(t))I_i && \text{for } 2 \leq i \leq 3, \qquad \text{(S1)} \\
\frac{\mathrm{d}}{\mathrm{d}t}J_i &= \gamma(1-\tau r(t))J_{i-1} + \mu(t)I_i - (\gamma(1-\tau r(t)) + m(t))J_i && \text{for } 2 \leq i \leq 3, \\
\frac{\mathrm{d}}{\mathrm{d}t}I_4 &= \gamma I_3 - (\gamma_{AIDS}(t) + \mu_{AIDS}(t))I_4, \\
\frac{\mathrm{d}}{\mathrm{d}t}J_4 &= \gamma(1-\tau r(t))J_3 + \mu_{AIDS}(t)I_4 - \gamma_{DAIDS}(t)J_4.
\end{aligned}
$$

This system of equations is also depicted in figure S6.

The parameters τ and $r(t)$ describe stage progression for those who are diagnosed and treated. The parameter $r(t) \in (0,1)$ reflects the estimated fraction of infected individuals for whom HAART is available provided clinical criteria for treatment are met. It is estimated independantly from the other parameters as described in section S2 .1. This fraction varies over time and is zero prior to 1995; it increases monotonically and plateaus in 2000. It is necessary to distinguish between the set of patients for whom HAART is potentially available and those who are effectively treated and have suppressed viral loads. Therefore, we introduce the extra parameter $\tau \in (0,1)$, which is time invariant, and controls how quickly treated individuals progress through infection. Diagnosed patients progress to the next stage of infection at a reduced rate modified by the factor $(1-\tau r(t))$. The product $\tau r(t)$ may be interpreted as the probability that HAART is available at time t and that viral loads are effectively suppressed. $r(t)$ is derived below.

S1 .1 Transmission & population genetic model

There are three necessary inputs into the population genetic model described in [8].

- Number of infected over time (eqns S1)
- Number of transmissions from each compartment to each compartment over time. This is called the 'birth' matrix $F(t)$.
- Number of transitions from each compartment to each compartment over time. This is called the 'migration' matrix $G(t)$.

Here we show how the latter two are calculated from the model.

Firstly, the migration matrix is deduced directly from the stage-progression and diagnosis terms in equations S1. For example, for undiagnosed chronic individuals in stage i, we have

$$G_{i,i+1}(t) = \gamma I_i.$$

And, if we order the 5 diagnosed stages after the 5 undiagnosed stages in the matrix, then the term

$$G_{i,i+5}(t) = \mu(t) I_i$$

captures the effects of diagnosis.

To construct $F(t)$, we must model the transmission rate from each compartment. Several parameters describe the relative contribution of infected individuals in different stages of infection and diagnosed/undiagnosed individuals to the total number of transmissions. These parameters are not identifiable from the timeseries data alone [5], and so by design, these parameters do not enter directly into the model for prevalence of infection over time. But these parameters are used in the population genetic model, and as shown in the main text, these parameters are identifiable from genetic data.

Given an incidence rate at some time, $\lambda(t)$, these parameters describe how many transmissions occur from each stage of infection and by diagnosis status. The equations developed here depend on, but do not contribute to, the equations that describe incidence and prevalence over time in the last section.

Transmissions attributable to undiagnosed infected individuals in stage i are proportional to the number infected in stage i and a weight β_i, which models differential contributions to the total number of transmissions from each stage. These weights are relative to undiagnosed EHI, which has $\beta_0 = 1$. The number of transmissions from an undiagnosed individual in stage i at time t will be proportional to the following weight:

$$\nu_i^I(t) = \beta_i I_i(t). \tag{S2}$$

$\delta \in (0, 1)$ is a time-invariant parameter which describes the relative infectiousness of diagnosed individuals upon learning that they are infected. It reflects changed behavior in response to diagnosis, such as serosorting and condom use.

The number of transmissions from a diagnosed individual in stage i at time t will be proportional to the following weight:

$$\nu_i^J(t) = \beta_i \delta J_i. \tag{S3}$$

β_i and δ are free parameters which are estimated as described in Text S2. We fix $\beta_0 = 1$, so that all other transmission rates are relative to undiagnosed EHI.

Once the rate of transmissions has been calculated from each compartment, the methods in [8] can be directly applied to calculate the likelihood of a genealogy given the solution of the compartmental model. We denote the transmsision rate of hosts in stage k and diagnosis status X to be $F_{kX}(t)$. This will be given by the product of total incidence

rate $\lambda(t)$ and the normalized weights ν.

$$F_{kX}(t) = \lambda(t) \frac{\nu_k^X(t)}{\sum_{i=0}^5 \nu_i^I(t) + \nu_i^J(t)}. \tag{S4}$$

As described above, this model has 6 free parameters (β_i for $i \in (0,4)$ and δ). We consider several simplifications. We combine chronic stages by defining the parameter $\beta_c := \beta_1 = \beta_2 = \beta_3$. The symbol β_a will be a synonym for β_4. We then consider several model variants:

- Free parameters: δ, β_c, β_a
- Free parameters: β_c, β_a. Fixed: $\delta = 1$.
- Free parameters: β_c, δ. Fixed: $\beta_a = \beta_c$.
- Free parameters: β_c. Fixed: $\delta = \beta_a = 1$.
- Free parameters: δ. Fixed: $\beta_c = \beta_a = 1$.

These models were independantly fitted to the data and compared by AIC.

S1 .2 Migration

A phylogeny estimated from HIV sequences from a given risk group defined by risk behavior and location is influenced by the epidemic both within and outside of the risk group. Lineages may both be imported and exported from the risk group. An ancestral lineage from the distant past is much more likely to represent an infected host outside of the risk group. For this reason, and since the model is specific to a single risk group, we include background HIV sequences from the LANL HIV database in order to determine which portion of the phylogenetic tree likely represents the epidemic in Detroit MSM.

We model immigration and emigration of lineages using a source/sink modification of the HIV model depicted in figure S8. Hosts emigrate to the sink population at a constant per capita rate ξ. We set the immigration from the source population to the risk group occurs at the same aggregate rate, so that the number of infected in the risk group remains unchanged and no direct modifications to the equations S1 are necessary. Once a lineage exits the Detroit MSM risk group, we assume there is zero probability that it will return or that it's descendants will return.

In the population genetic model, the following modification is required to account for migration. Let index k correspond to the source population. Then,

- $G_{k,i}(t) = \xi I_i(t)$ if i corresponds to an undiagnosed category or $G_{k,i}(t) = \xi J_i(t)$ if i corresponds to a diagnosed category.
- Letting $Y_k(t)$ denote the size of the source population, we use $Y_k(t) = 400 \times \sum_i I_i + J_i$. The factor of 400 was chosen so that $Y_k \approx 1.2 \times 10^6$ in the present day.
- $F_k(t) = 400\lambda(t)$ describes the number of transmissions within the source population.

The parameter ξ is a free parameter that is estimated from the genetic data as described below.

S2 Natural history

Data from the Multicenter AIDS Cohort Study (MACS) [9] was used to parametrize models of chronic infection. We analyzed 190 patients with known dates of seroconversion and AIDS before the availability of HAART. EHI durations were drawn from an exponential distribution (mean one year) which gave many replicates of chronic infection intervals consistant with the MACS data. An Erlang distribution was fitted to these simulated

intervals. The shape parameter was selected by maximum likelihood (*fitdistr* in R). The compartmental model with three chronic stages of duration 2.1 years was selected to reproduce this distribution.

The distribution of the duration of chronic infection is shown in figure S9. The duration of AIDS in the model varies over time and depends on the empirical mortality rates from the surveillance data.

S2 .1 Availability of HAART

This section discusses the derivation of $r(t)$, the availability of HAART given clinical requirements for treatment are satisfied. This should not be interpreted as the proportion of individuals treated and with suppressed viral loads at time t; that is described by the product $\tau r(t)$. HAART became available around 1995 and its availability to diagnosed individuals increased steadily over the next 5 years. To model the increasing availability of HAART, we used data collected in Michigan from the Medical Monitoring Project (MMP) [10]. MMP provided information on 162 individuals currently in treatment in Michigan, including the date of first positive HIV test and the date of first antiretroviral usage. Data from MMP is illustrated in figure S10. In 1995, there were 23 people previously diagnosed and 7 in treatment (30%). In 2000, there were 66 people previously diagnosed and 51 of which were in treatment (77%).

The time from diagnosis to treatment was assumed to be Weibull distributed. This interval was modeled using a parametric survival regression (*survreg* in R) adjusting for the year of diagnosis and assuming right-censoring at the time of the MMP survey. It was assumed that availability of HAART plateaued in year 2000.

$F_s^{\text{HAART}}(\Delta t)$ will denote the fraction of diagnosed infected individuals on HAART Δt years after diagnosis among those individuals diagnosed at time s. This fraction is given

by the CDF of the Weibull distribution with parameters from the survival regression model. Supposing that HAART availability does not increase for infected cohorts beyond $s = 2000$, we calculate the availability of HAART in year t by averaging the fraction on HAART for those diagnosed up to 7 years in the past relative to the the fraction on HAART for the infected cohort in 2000. $r(t) = 0$ for $t < 1995$. For $t \geq 1995$, we have

$$r(t) = \frac{1}{7} \int_{s=(t-7)}^{t} \frac{F_s^{\text{HAART}}(t-s)}{F_{2000}^{\text{HAART}}(t-s)} \mathrm{d}s \quad \text{if } t \geq 1995.$$

The estimated $r(t)$ is illustrated in figure S10.

Supplementary References

1. Prejean J, Song R, Hernandez A, Ziebell R, Green T, et al. (2011) Estimated HIV incidence in the United States, 2006–2009. PLoS One 6: e17502.

2. Cohen M, Shaw G, McMichael A, Haynes B (2011) Acute HIV-1 infection. N Engl J Med 364: 1943–1954.

3. Hallett T, Ghys P, Bärnighausen T, Yan P, Garnett G (2009) Errors in BED-derived estimates of HIV incidence will vary by place, time and age. PLoS One 4: e5720.

4. Parekh B, Hanson D, Hargrove J, Branson B, Green T, et al. (2011) Determination of mean recency period for estimation of HIV Type 1 incidence with the BED-capture EIA in persons infected with diverse subtypes. AIDS Research and Human Retroviruses 27: 265–273.

5. Bezemer D, de Wolf F, Boerlijst M, van Sighem A, Hollingsworth T, et al. (2010) 27 years of the HIV epidemic amongst men having sex with men in the Netherlands: An in depth mathematical model-based analysis. Epidemics 2: 66–79.

6. Hogan D, Zaslavsky A, Hammitt J, Salomon J (2010) Flexible epidemiological model for estimates and short-term projections in generalised HIV/AIDS epidemics. Sex Transm Infect 86: ii84.

7. Eilers P, Marx B (1996) Flexible smoothing with B-splines and penalties. Statistical Science : 89–102.

8. Volz E (2012) Complex population dynamics and the coalescent under neutrality. Genetics 190: 187–201.

9. Kaslow R, Ostrow D, Detels R, Phair J, Polk B, et al. (1987) The Multicenter AIDS Cohort Study: rationale, organization, and selected characteristics of the participants. American Journal of Epidemiology 126: 310.

10. McNaghten A, Wolfe M, Onorato I, Nakashima A, Valdiserri R, et al. (2007) Improving the representativeness of behavioral and clinical surveillance for persons with HIV in the United States: the rationale for developing a population-based approach. PLoS One 2: e550.
