# Supplementary material for: HIV-1 Transmission during Early Infection in Men Who Have Sex with Men: A Phylodynamic Analysis
Source: PLoS Med. 2013 Dec 10;10(12):e1001568. doi: 10.1371/journal.pmed.1001568 (PMC3858227; doi:10.1371/journal.pmed.1001568)
Supplement: Text S3 — Model validation and simulations. (PDF) [file pmed.1001568.s023.pdf]

Supporting methods for *HIV-1 Transmission During Early Infection in Men Who Have Sex with Men: A Phylodynamic Analysis*

Erik M Volz*[1], Edward Ionides[2], Ethan Romero Severson[3], Mary Grace Brandt[4], Eve Mokotoff[4], James S Koopman[5]

* Corresponding author: e.volz@imperial.ac.uk

1. Department of Infectious Disease Epidemiology, Imperial College London, UK

2. Department of Statistics, University of Michigan- Ann Arbor, USA

3. Theoretical Biology and Biophysics, Los Alamos National Laboratory, Los Alamos,New Mexico

4. Michigan Department of Community Health, Detroit, MI, USA

5. Department of Epidemiology, University of Michigan- Ann Arbor, USA

September 27, 2013

S1 Simulations and sensitivity analysis

S2 Model variations and model comparisons

Simulation results in section S2 .2 are based on a slightly different model of the natural history of infection then the one used for the main results. This model more closely

resembles the model in [1] and reuses several parameter values from that study. Infection progresses through five stages of equal average duration 1.89 years. The rate of progression from stage i to $i+1$ is $\gamma = 1/1.89$ year^{-1}. This model yields a Gamma-distributed time interval from infection to AIDS, and was found to accurately reproduce the time to AIDS for a Dutch cohort in [1]. We will sometimes refer to stage 5 as "late HIV infection", which includes individuals with AIDS.

The diagnosis rate in stage 5 is given by the rate μ_{AIDS}, which represents increased rates of diagnosis when patients have AIDS symptoms. Following [1], we fix $\mu_{\text{AIDS}} = 12/365$ days

Mortality from natural sources occurs at the rate $m = 1/40.28$ year^{-1}. This rate was based on US Census data for SE Michigan and accounting for the average age at diagnosis.

S2 .1 Sensitivity to demographic stochasticity

A simulation experiment was carried out with the following aims

- Determine sensitivity of estimates to stochastic population dynamics in contrast to the deterministic dynamics used in the HIV model (Text S1).
- Determine sensitivity of estimates to the possibility that sampled individuals have descendants that are themselves sampled. This is an effect which is not captured by the coalescent model.
- Establish identifiability of parameters β_c and δ which respectively describe the relative infectiousness of those with chronic HIV infection and those that are diagnosed. These parameters are estimated directly from the HIV phylogeny.

A continuous-time/individual-based simulation was carried out with rates given by the solution of the deterministic HIV model (see Text S1). The procedure is as follows:

1. The deterministic model was solved with parameters at the MLE (see main text).
2. Rates of birth and migration ($F(t)$ and $G(t)$) were abstracted from the model solution. Deaths (natural and AIDS) were also calculated.
3. Discrete simulations using the Gillespie algorithm were carried out using the given rate matrices and death rates.
4. A genealogy was abstracted from the simulation by keeping track of which unit transmitted to each other unit.
5. A sample was collected to replicate our real data. $n = 437$. Times of sampling and the stage of infection of each sample unit were matched to real data.

The coalescent likelihood (see Text S2, [2]) was then solved using the deterministic model over a range of β_c and δ parameters. The results of these calculations are shown in figure S13. We conclude:

- The relative infectiousness of chronic and diagnosed individuals is identifiable under the conditions described in the analysis presented in the main text.
- Demographic stochasticity can bias estimated transmission rates using a deterministic model, but the bias should not be great in absolute terms. For example, in this experiment, bias in β_c was approximately 0.05 on a scale of zero to one. It is also possible that sampling of direct descendants of those who are already sampled may bias the estimates, but this bias was not large in this experiment.

S2 .2 Sensitivity to phylogenetic error

Since the coalescent model is fitted to phylogenies estimated separately, parameter estimates are subject to error in estimated phylogenies. Estimation of phylogenetic branch

lengths can be imprecise. Sequence evolution includes large variation in mean substitution rates across sites; parameters describing this variation must be estimated, reducing power to estimate the mean mutation rate. Approximately half of sites are invariant, leaving relatively few substitutions per branch on which to estimate chronological branch lengths. A simulation experiment was carried out to asses the robustness of our estimation procedure to realistic levels of phylogenetic error. The experiment is illustrated in figure SS14 and consisted of the following steps:

1. The *true* vector of parameters θ were selected. We used $\beta_1 = .5$ and $\beta_i = .1$ for $i > 1$; $\delta = .1$.

2. A coalescent tree $\mathcal{G}$ was simulated using the methods described in [2]. This method simulates a gene genealogy that is consistent with a given timeseries of disease prevalence, transmissions, and stage-transitions. Code to conduct these simulations are available at *http://code.google.com/p/colgem/*.

3. A set of 409 sequences $\mathcal{S}$ was simulated on $\mathcal{G}$ using *seq-gen* [3] (*http://tree.bio.ed.ac.uk/software/seqgen/*).
   Sequences were 960 characters and generated with an HKY85 nucleotide substitution model. Parameters of this model were drawn from an ML phylogeny (phyml) estimated from the Detroit sequences. To mimic the actual data collection, the following sample sizes were collected at 4 time points at 2 year intervals from present to past: (307, 77, 19, 6). Sampling infected in stage i was in proportion to the prevalence of stage i.

4. A phylogeny $\hat{\mathcal{G}}$ was estimated from the simulated sequences using BEAST under the similar conditions as those described above (5 independent chains, HKY model).

5. An estimate of the parameters $\hat{\theta}$ was obtained using the coalescent likelihood described in [2]. Model fitting was conducted using an iterated importance sampling method described in [4].

Model fitting in the last step was done using Incremental Mixture Importance Sampling (IMIS), a Bayesian adaptive importance sampling method which iteratively adapts the sampling distribution to the posterior [4]. IMIS has previously been shown to work well when fitting complex models of HIV transmission to timeseries [5]. IMIS begins by sampling $d \times 1000$ particles from the multivariate prior distribution of model parameters where d is the number of parameters fitted. Subsequent generations of the importance sampler generate $d \times 100$ particles from a mixture of multivariate normal distributions centered on the particle with the highest importance weight in the previous generation. The process terminates when the proposal distribution is close to uniform. The posterior was approximated by sampling 3000 particles from the final generation.

In order to improve computational efficiency of this method, the priors were modeled with a mixture of multivariate normal distributions which were pre-adapted to the log likeihood $\mathcal{L}(\mathcal{T})$. This prevented the sampling of any particles with IMIS which would have vanishingly small likelihood. This precaution was taken because calculation of the coalescent log likelihood $\mathcal{L}(\mathcal{G})$ is computationally expensive. The ODE model in Text S1 was fitted 2700 times by maximizing $\mathcal{L}(\mathcal{T})$ from a random starting condition (uniformly distributed). Maximization used the Nelder-Mead method (*optim* in R [6]). Each of 2700 parameter estimates based on likelihood maximization was used as the basis for a prior distribution, which was modeled as a mixture of multivariate normal distributions (*mclust* in R [7]). Uniform priors were used for transmission parameters β_i.

Figure SS14 shows estimated ($\hat{\mathcal{G}}$) versus true ($\mathcal{G}$) external branch lengths. BEAST does a good job of estimating the relative length of branches in $\mathcal{G}$, but in some replicates,

the mean rate was misspecified, causing branch lengths to be systematically over- or under-estimated (results not shown). In these instances, we add a calibration step to the coalescent likelihood: Prior to calculating the likelihood, branch lengths are rescaled so that TMRCA predicted by the coalescent model coincides with the TMRCA in the phylogeny. The set of *ancestor* equations [8] describing the coalescent process in the epidemiological model were solved, which provided a prediction for the number of lineages through time from the present to the beginning of the epidemic. Each posterior tree was rescaled to match the time at which there were 50 lineages in the coalescent model.

Figure SS15 shows the results of three model fits to different trees estimated with BEAST. The parameters β_1 and β_{25} are shown, which respectively describe the contribution of early chronic infection and late chronic infection to total transmissions. In all cases the 95% confidence intervals covers the median value estimated from the true simulated coalescent tree; and, the estimates are close to the parameter values used to generate the coalescent tree. This demonstrates that it is possible to distinguish transmission rates during EHI from early chronic infection and from late chronic infection.

S2 .3 Sensitivity to violation of coalescent model assumptions

The coalescent model used to generate the results in the main text was based on the assumption that the time of an internal node in an HIV phylogeny corresponds to the time of a transmission event. In reality, a population of virus with a large diversity of unique haplotypes circulates within hosts, and the variant which is transmitted may differ substantially from the predominant variants within a host. The TMRCA estimated in a phylogeny may not correspond exactly to the time of transmission, but rather to the TMRCA for a sampled variant and a transmitted variant.

We did a simulation experiment with the following aims:

- Establish a plausible distribution of intra-host coalescent times.
- Assess the amount of bias that is likely to occur in our fitted models if nodes in the tree correspond to intra-host coalescent times rather than transmission events.

The simulation experiment protocol is as follows:

1. **Distribution of intra-host coalescent times.** We used phylogenies estimated in [9] for nine patients described in [10] (subsequently referred to as the *Shankarappa data*). In [10], nine patients with known seroconversion dates were followed and virus was sampled at regular intervals. Sequencing of *env* was done using the SGA method. In [9], relaxed clock phylogenies were estimated using the known sample dates, yeilding branch lengths in units of calendar time. For each patient, and for each time the patient was sampled, we calculated TMRCA for all pairs of sequences sampled at that time.

2. We carried out a discrete-event individual-based simulation of the HIV model as described in section S2 .1 with the following modifications. Following a transmission event or a sampling event, there are two daughter lineages i and j and an ancestral node α. We perturb the time of node α backwards in time by Δt, which is drawn from the empirical distribution of intra-host coalescent times calculated in step 1. This distribution also depends on how long the transmitting node has been infected. Specifically, we find the time-to-seroconversion and corresponding coalescent times in the Shankarappa dataset that most closely matches how long the simulated individual has been infected; then we draw a random intra-host coalescent time at random from the set of coalescent times in that sample.

3. A random sample of 437 individuals is taken at times which match those of the real

dataset described in the main text. The states of these sampled individuals are also chosen to match the empirical data.

4. We calculate the coalescent likelihood (see Text S2) over a range of β_c and δ parameters. Parameters controlling incidence are fixed at the MLE since those parameters are mostly determined by the surveillance timeseries $\mathcal{T}$, and β_c and δ are only estimated from the genetic data.

Figure S S16 shows the distribution of intra-host coalescence times for isochronously sampled sequences. Figure S S16 also shows how this distribution depends on the time since seroconversion that samples are taken. There is an almost linear increase in the median TMRCA as a function of time since seroconversion. Median TMRCA is about 35% of time to seroconversion, however the distribution is highly bi-model, with many small TMRCAs and a few deep branches in the tree generate large TMRCAs.

If we compare the simulated tree in this experiment with the one generated in section S2 .1 (based on node time = transmission time), we see that there is not a very large difference in branch lengths. The median branch length in this simulated tree is 1338 days versus 1224 days in the other tree– a bit longer as would be expected since node heights are perturbed backwards in time. However a KS test does not show a significant difference in branch lengths of the two trees (p=43%).

Figure S17 shows the likelihood surface computed from a mesh of β_c and δ parameters. These parameters describe the relative infectiousness of chronic infections and diagnosed individuals. The maximum likelihood occurs close to the true parameters values.

It may seem surprising that perturbing node heights by the distribution in figure S16 does not have a more drastic impact on the likelihood surface. This occurs for a couple reasons. A large proportion of transmissions are from those who have not been infected

very long, and consequently the absolute difference in the time of transmission and intra-host coalescent time is not very great. Because of this, the median change in branch lengths in the simulated tree is only 78 days and the mean is 300 days. On the other hand, large changes in branch lengths occurs for people who transmit after being infected for a long time, and those individuals are likely to be connected to the tree by long branch lengths anyways.

S3 Dual infection

This section provides a simple approximation to the bias in coalescence rates that may occur from neglecting the effects of dual-infection (including co-infection and super-infection) [11].

Consider a transmission event from a donor A to a recipient B. With probability q, A is dual-infected. The abundance of the dual-infecting strain i in A is p and $1-p$ for the strain j which initiated infection in A. Given that A harbors virus that is ancestral to the sample, we will make the approximation that the strain i is ancestral with probability p and j is ancestral with probability $1-p$. This is a conservative approach, since there is the possibility that both i and j are ancestral. Upon transmission from A to B, the probability of a coalescent event is

$$c = (1-q) + q(p^2 + (1-p)^2). \tag{S1}$$

Supposing prevalence of dual-infection among transmitting hosts is $q = 10\%$ and abundance of strain i is $p = 25\%$, we have $c = 96.25\%$ (the probability is $c = 1$ in the absence of dual infection). When fitting the coalescent model, the coalescent probability is less than anticipated, so the estimated incidence would be biased downwards to compensate.

In this case, incidence would be biased downwards by about $1 - c = 3.75\%$.

It is also important to consider that even if the transmission A→ B does not result in a coalescent (with probability $1-c$), the coalescent event will eventually happen, and will probably coincide with the time that A became dual-infected. Dual infection therefore has the effect of delaying time to coalescence. If this delay in coalescent times is not great, estimated incidence will probably have a bias much less than $1 - c$.

S4 CD4 and model validation

A simple least squares model of mean CD4 count was used to validate the MLE incidence, prevalence, and diagnoses from each stage of infection. Denote the MLE number of diagnoses from stage k in year i as X_{ik}. The matrix X has dimensions $T \times 3$ where T is the number of years for which we have aggregated CD4 counts and the stage of infection is EHI, chronic, or AIDS. The estimated mean CD4 count for each stage is encoded in the vector $\mathbf{a} = (a_0, a_1, a_2)$. The estimated mean CD4 count for new diagnoses over time is given by the product $\hat{Y} = X \cdot \hat{a}^\top$. The actual mean CD4 count at each time step is given by the column vector Y. We have estimated the vector $\hat{a}$ which minimizes the weighted residual sum of squares

$$\sum_{i=1}^{T}(n_i Y_i - n_i \hat{Y}_i)^2 = \sum_{i=1} n_i^2 (Y_i - X \cdot \hat{a}^\top)^2,$$

where n_i is the number of CD4 counts measured in timestep i. The estimated mean CD4 $\hat{Y}$ is shown in figure 3B in the main text and the vector $\hat{a}$ is shown in the inset.

S5 Simulated trees

To give greater intuition for why variation in transmission rates is identifiable from genetic data, we have simulated gene trees under scenarios where

- All infected individuals transmit at the same (time-dependent) rate (figure S18).
- EHI transmit at a rate in excess of chronic infections as described by the MLE fit of the model described in the main text (figure S18).

We also show the HIV phylogeny for comparison (figure S18). Trees were simulated using the methods described in [2] and available at *http://code.google.com/p/colgem/*. The times and states of patients in the simulated trees were chosen to match the real data.

As mentioned in the main text:

> There is substantial molecular epidemiological evidence that variation in transmission rates over the course of infection influences the genetic diversity of HIV [12–15]. For example, viral sequences isolated from patients who were recently infected tend to be phylogenetically clustered (more closely related to one another than expected by chance). Simple models of HIV transmission have been shown to reproduce these phylogenetic patterns [16], suggesting that the transmission rate from EHI could be identifiable from genetic data.

In addition to the results presented in [16], these simulations give a graphical representation of how EHI transmission influences HIV phylogenetic structure.

Supplementary References

1. Bezemer D, de Wolf F, Boerlijst M, van Sighem A, Hollingsworth T, et al. (2010) 27 years of the HIV epidemic amongst men having sex with men in the Netherlands: An in depth mathematical model-based analysis. Epidemics 2: 66–79.

2. Volz E (2012) Complex population dynamics and the coalescent under neutrality. Genetics 190: 187–201.

3. Rambaut A, Grass N (1997) Seq-Gen: an application for the Monte Carlo simulation of DNA sequence evolution along phylogenetic trees. Computer Applications in the Biosciences: CABIOS 13: 235–238.

4. Raftery AE, Bao L (2010) Estimating and projecting trends in HIV/AIDS generalized epidemics using incremental mixture importance sampling. Biometrics 66: 1162–1173.

5. Hogan D, Zaslavsky A, Hammitt J, Salomon J (2010) Flexible epidemiological model for estimates and short-term projections in generalised HIV/AIDS epidemics. Sex Transm Infect 86: ii84.

6. R Core Team (2012) R: A Language and Environment for Statistical Computing. R Foundation for Statistical Computing, Vienna, Austria. URL `http://www.R-project.org/`. ISBN 3-900051-07-0.

7. Fraley C, Raftery A (2006) MCLUST version 3 for R: Normal mixture modeling and model-based clustering. Technical report.

8. Volz E, Pond S, Ward M, Leigh Brown A, Frost S (2009) Phylodynamics of infectious disease epidemics. Genetics 183: 1421–1430.

9. Lemey P, Pond SLK, Drummond AJ, Pybus OG, Shapiro B, et al. (2007) Synonymous substitution rates predict HIV disease progression as a result of underlying replication dynamics. PLoS Comput Biol 3: e29.

10. Shankarappa R, Margolick J, Gange S, Rodrigo A, Upchurch D, et al. (1999) Consistent viral evolutionary changes associated with the progression of human immunodeficiency virus type 1 infection. J Virol 73: 10489.

11. Redd A, Mullis C, Serwadda D, Kong X, Martens C, et al. (2012) The rates of HIV superinfection and primary HIV incidence in a general population in Rakai, Uganda. J Infect Dis 206: 267–274.

12. Pao D, Fisher M, Hué S, Dean G, Murphy G, et al. (2005) Transmission of HIV-1 during primary infection: relationship to sexual risk and sexually transmitted infections. AIDS 19: 85.

13. Yerly S, Junier T, Gayet-Ageron A, Amari E, von Wyl V, et al. (2009) The impact of transmission clusters on primary drug resistance in newly diagnosed HIV-1 infection. AIDS 23: 1415.

14. Cuevas M, Muñoz-Nieto M, Thomson M, Delgado E, Iribarren J, et al. (2009) HIV-1 transmission cluster with T215D revertant mutation among newly diagnosed patients from the Basque Country, Spain. JAIDS J Acquir Immune Defic Syndr 51: 99.

15. Aldous J, Pond S, Poon A, Jain S, Qin H, et al. (2012) Characterizing HIV Transmission Networks Across the United States. Clin Infect Dis 55: 1135–1143.

16. Volz E, Koopman J, Ward M, Brown A, Frost S (2012) Simple epidemiological dynamics explain phylogenetic clustering of HIV from patients with recent infection. PLoS Comput Biol 8: e1002552.
