# Supplementary material for: HIV-1 Transmission during Early Infection in Men Who Have Sex with Men: A Phylodynamic Analysis
Source: PLoS Med. 2013 Dec 10;10(12):e1001568. doi: 10.1371/journal.pmed.1001568 (PMC3858227; doi:10.1371/journal.pmed.1001568)
Supplement: Text S4 — Detailed methods used for model fitting and parameter estimation. (PDF) [file pmed.1001568.s024.pdf]

Supporting methods for *HIV-1 Transmission During Early Infection in Men Who Have Sex with Men: A Phylodynamic Analysis*

Erik M Volz*[1], Edward Ionides[2], Ethan Romero Severson[3], Mary Grace Brandt[4], Eve Mokotoff[4], James S Koopman[5]

* Corresponding author: erikvolz@umich.edu

1. Department of Infectious Disease Epidemiology, Imperial College London, UK

2. Department of Statistics, University of Michigan- Ann Arbor, USA

3. Theoretical Biology and Biophysics, Los Alamos National Laboratory, Los Alamos,New Mexico

4. Michigan Department of Community Health, Detroit, MI, USA

5. Department of Epidemiology, University of Michigan- Ann Arbor, USA

September 27, 2013

S1 Estimating stage of infection

Stage of infection (0-4) was estimated on the basis of CD4 at diagnosis, date of last negative test, ambiguous sites in the HIV sequence, and concurrent diagnosis with AIDS.

Instead of using incidence assays based on differential antigen/antibody assays, we

used the frequency of ambiguous sites (FAS) within DRM sequences to detect diagnoses with EHI. A high FAS is an indicator of a diverse intra-host viral population resulting from a long period of intra-host evolution. FAS has previously been shown to be highly informative about the recency of infection in the individual from whom virus was sampled [1,2]. An advantage of this approach is that a customized threshold FAS value was found to have a high positive predictive value for detecting EHI, which we define as the first year of infection. We defined EHI to be one year rather than the more commonly used periods of 2 or 6 months because the simulation studies described in section Text S4 revealed that transmission rates for shorter periods were not identifiable given the available number and quality of HIV sequences.

Our goal for using FAS is different than that of [1,2], who were focused on developing a test with good sensitivity and specificity that could be useful for incidence estimation. Our objective is to design a test with high positive predictive value for identifying EHI (1st year of infection), which allows us to select sequences for inclusion in the phylogenetic analysis which are highly likely to have come from a true EHI. Given the data available to us, we find that such a test is possible, though it has poor sensitivity.

CD4 counts collected more than 6 months from the diagnosis date were excluded. AIDS diagnosis was considered to be concurrent with HIV diagnosis if AIDS diagnosis was within 2 months of the first positive HIV test.

The square root of CD4 counts are known to decrease approximately linearly over the course of infection [3]. Figure S11 shows the distribution of root CD4 from the Michigan surveillance data, only considering cases known to be EHI or AIDS. Using these surveillance data, we fit a linear model $\sqrt{\mathrm{CD4}} = i + \epsilon$, where $i \in [0, 4]$ is the stage of infection. Only patients who were known to be either EHI or AIDS were used when fitting the model. The root CD4 values and fitted model are shown in figure S11.

A test for EHI with a high PPV of 86% was developed based on FAS [1,2]. To develop this test, we examined the set of MDCH sequences which were definitely from EHI based on the date of last negative test. 643 sequences were definitely EHI, while 498 were definitely from late infection. Figure S12 shows the probability and odds that a sequence originated from EHI given the threshold FAS value. We used the highest PPV threshold of 3 ambiguous sites.

A *naive Bayes* model was developed to derive the probability $P_u(i|\theta_u)$ that patient u is in stage i at the time of diagnosis conditional on a vector of clinical covariates θ_u. Covariates consisted of CD4 counts within 6 months of diagnosis x, FAS, and whether the patient was diagnosed with AIDS within two months of HIV diagnosis. Naive Bayes models are easy to fit and the results are easily interpretable, but they depend on the approximation that variables used for classification are conditionally independent. There is undoubtedly some dependence among variables such as CD4 and FAS which is not captured by this model. But, naive Bayes classifiers tend to perform well unless this dependence is near that between the objective variable (stage of infection in this case) and the predictor variable [4]. It would be optimal, although impractical in the current setting and with available data, to develop a model for the joint distribution of all predictor variables and the objective variable.

Let θ_u be a vector of p covariates for patient u that will be used to estimate the stage of infection. We will estimate $P(i|\theta_u)$, the probability that u is in state i at diagnosis. Using Bayes' rule

$$P(i|\theta_u) = \frac{P(i)}{P(\theta_u)} P(\theta_u|i). \tag{S1}$$

In practice, $P(\theta_u|i)$ can be difficult to calculate, so naive Bayes models make the approx-

imation

$$P(i|\theta_u) \approx \frac{P(i)}{C} \prod_{k=1}^{p} P(\theta_{uk}|i), \tag{S2}$$

where C normalizes the pmf. Then we only need to calculate the conditional probabilities $P(\theta_{uk}|i)$ for the following CD4, FAS, and AIDS: $P(\sqrt{\text{CD4}}|i)$ is given by the normal distribution fitted by the linear regression model above.

We will use the indicator function $I_{FAS3}(u)$ which takes a value of 1 if FAS< 3 and is zero otherwise. $P(\text{FAS}|i)$ is found by cross-tabulating I_{FAS3} and the known EHI status of infected individuals. These data are arranged in the following confusion matrix:

	$I_{FAS3} = 1$	$I_{FAS3} = 0$
$i = 0$	$A = 171$	$B = 472$
$i > 0$	$C = 21$	$D = 477$

Then, for example,

$$P(I_{FAS3} = 1|i = 0) = A/(A + B) = 27\% \tag{S3}$$

$$P(I_{FAS3} = 0|i = 0) = B/(A + B) = 73\% \tag{S4}$$

$$P(I_{FAS3} = 1|i > 0) = C/(C + D) = 4\% \tag{S5}$$

$$P(I_{FAS3} = 0|i > 0) = D/(C + D) = 96\% \tag{S6}$$

$P(\text{AIDS}|i)$ is straightforward, since detection of AIDS can reasonably be approximated to have perfect sensitivity and specificity. Let $I_{\text{AIDS}}(u)$ be the indicator function for

whether a patient has AIDS.

$$P(I_{\text{AIDS}} = 1|i = 4) = 1 \tag{S7}$$

$$P(I_{\text{AIDS}} = 0|i = 4) = 0 \tag{S8}$$

$$P(I_{\text{AIDS}} = 1|i < 4) = 0 \tag{S9}$$

$$P(I_{\text{AIDS}} = 0|i < 4) = 1 \tag{S10}$$

Applying these conditional probabilities to equation S2 gives our estimate of $P(i|\theta_u)$. The prior $P(i)$ is based on the number of infections in each stage in 2011 from a MLE fit of the HIV model to timeseries data alone (section S3).

S2 Coalescent model

An epidemiological coalescent model [5–9] was derived from the transmission model using the methods described in [9]. Code used to calculate the likelihood of a tree using epidemiological coalescent model is available at *http://code.google.com/p/colgem/*.

The coalescent model provides a means of calculating the likelihood of a gene genealogy conditional on a complex demographic history. The time series consists of the prevalence of diagnosed and undiagnosed infections in each stage, the number of transmissions by each stage, and the flux of infected individuals between states (by diagnosis or disease progression). The time series data were aggregated into 473 intervals. The likelihood is also a function of states of the patients from whom the HIV sequences were sampled (see section S1). Estimation was carried out using the mean likelihood from a random sample 10 trees from the BEAST posterior (see Text S3).

The coalescent likelihood of a gene genealogy $\mathcal{G}$ has the form

$$P(\mathcal{G}) = \prod_{(i,j,s_\alpha)\in\mathcal{G}} q_{ij}(s_\alpha), \tag{S11}$$

where $\mathcal{G}$ is the set of nodes in the genealogy, i and j are lineages that coalesce at a node at time s_α, and $q_{ij}(s_\alpha)$ is the rate that lineages i and j coalesce times the probability that they have not yet coalesced at time s_α. In [9], it is shown how to derive s_α and q_{ij} from the 'birth' and 'migration' matrices described in Text S1.

S3 Model fitting

The ODE model equations were fitted to the surveillance data $\mathcal{T}$. The surveillance data consisted of all annual diagnoses, the number of diagnoses concurrent with AIDS, and the number of AIDS diagnoses among patients previously diagnosed with HIV. The likelihood of the surveillance data was derived under the condition that the number of reported diagnoses is Poisson distributed around the values predicted by the deterministic ODE model.

Let $y(t)$ be the aggregated number of diagnoses in year t, $y_A(t)$ be the aggregated number of diagnoses concurrent with AIDS diagnosis, and $y_{CA}(t)$ be the aggregated number of AIDS diagnoses among previously diagnosed HIV cases. $\bar{y}$ will represent the corresponding quantities from a solution of the HIV model. The likelihood is

$$P(\mathcal{T}) = \prod_t P_{\text{Pois}}(y(t)|\bar{y}(t))P_{\text{binom}}(y_A(t)|\bar{y}(t), \bar{y}_A(t))P_{\text{Pois}}(y_{CA}(t)|\bar{y}_{CA}(t)) \tag{S12}$$

The important effect of including AIDS diagnoses in the model and the likelihood is to constrain the tradeoff between incidence and the observed total number of diagnoses.

Similarly, by modeling the likelihood of $y_{CA}(t)$ AIDS diagnoses from previously diagnosed individuals, we fit the model to trajectories that closely match the known number of diagnosed AIDS cases over time. By constraining the number of diagnosed AIDS cases, this may increase the identifiability of the stage progression parameter τ.

With the log likelihoods $\mathcal{L}(\mathcal{T})$, the ODE model equations were fitted by maximum likelihood. The likelihood was maximized using the simplex method in R (Nelder-Mead *optim*), with periodic re-starts and stochastic search.

1. 6000 random starting conditions are drawn uniformly from a $p-$dimensional parameter space. Each of these will be called a *particle*.
2. While the MLE has not been found:
   (a) The likelihood is optimized for each particle for 200 iterations.
   (b) If there is no improvement in the maximum likelihood particle: terminate.
   (c) The top 10% of 6000 particles are retained. The remaining 90% of particles were drawn from a multivariate normal distribution with center chosen independantly and uniformly at random from the top 10% of particles. The variance covariance matrix was calculated from the top 10% of particles.

Convergence was tested by running the optimizer twice and ensuring the same MLE was reached.

Given an MLE fit of the model, the parameters β_c, β_a and ξ (see section Text S1) were estimated from the genetic data using the likelihood equation S11. The simplex method was used as above, but since there are only 3 parameters, optimization was started from 18 random initial conditions and we did not use periodic re-starts with stochastic search. All solutions were checked for convergence and consistency. Likelihood profiles and 95% con-

fidence intervals were constructed for each parameter $\theta = (\beta_c, \beta_a, \xi)$. Deriving confidence intervals for derived quantities, such as the proportion of transmissions from EHI over time is more challenging, and likelihood profiles cannot be easily translated to estimated transmission fractions. We used an approach which is formally an empirical Bayes approximation to the confidence interval [10]. This approach uses a prior distribution which is calculated directly from the data. In our case, the prior is based on the likelihood profiles; we constructed a multivariate uniform prior with bounds given by the 97.5% CIs calculated for each parameter using the profile method. A Bayesian sampling-importance-resampling method was then used to sample epidemic trajectories from the multivariate uniform prior. One thousand parameter vectors were sampled from the posterior yielding 938 unique trajectories.

S3 .1 Overdispersion

We have analyzed the predicted and actual diagnoses aggregated by year for evidence of overdispersion as well as suitability of the Poisson distribution more generally. There is evidence for a few outliers in the early epidemic, but overall the Poisson model holds up quite well. We can quantify overdispersion using the statistic

$$R = \frac{1}{n} \sum_i z_i^2$$

where n is the number of years, and z_i is the Z-score corresponding to the predicted and actual number of diagnoses in the i'th year. The statistic R will asymptotically follow a χ^2 distribution with n degrees of freedom. Using all years of data, we find $R = 2.48$, indicating moderate overdispersion. Removing the early outliers (first 10 years of the epidemic), we find $R = 1.24$ and $p = 0.21$ (χ^2 test). Because outliers occur early in the

epidemic, estimated incidence will not be biased close to the present, and including extra parameters to cope with overdispersion would be unwarranted.

We also examined the suitability of the Poisson distribution using a KS test of the CDF of the residuals. This test verifies that the residuals follow a Poisson distribution with variable rates and gives an indication of the suitability of the model over all quantiles, not just the tails. Note that we cannot do a direct test of residuals against a Poisson distribution, because each data point has a distinct rate parameter; therefore we compare the CDF of residuals to a uniform distribution. In this case, we find $D = 0.17, p = 0.29$ without removing outliers.

S3 .2 Computation

In this study, we were limited to using only about a quarter of potentially informative sequences by the computational demands of both estimating a relaxed clock phylogeny and in fitting complex models to the estimated phylogeny. The phylogenetic algorithms implemented in BEAST are $O(n^2)$ in the number of sequences n. Calculation of the coalescent likelihood [9] is $O(n)$ in the number of sequences and $O(m^2)$ in the number of states of the transmission model. The computational requirements of fitting the HIV transmission system model are more sensitive to the complexity of the model than to the sample size. Estimation of the phylogeny was carried out in parallel on 9 multicore processors (10 independent Markov chains) with 12 cores each and using the BEAGLE library [11]. Calculating a single coalescent likelihood with a single tree of 762 taxa takes about 30 seconds with a 2GHz processor. The computational complexity is highly sensitive to the complexity of the model; models with fewer states could be fitted more easily, but might not capture essential features of the natural history of HIV or the effects of diagnosis on transmission. Fitting a model requires tens of thousands of likelihood calculations, and

the likelihood must be averaged across a sample of trees from the Bayesian-phylogenetic posterior. ML estimation with the coalescent likelihood was carried out in parallel on a cluster with 200 nodes. Fitting all model variants described in section Text S1 and estimating confidence intervals using a sample of 10 trees took about one week on this cluster.

Supplementary References

1. Kouyos R, von Wyl V, Yerly S, Böni J, Rieder P, et al. (2011) Ambiguous nucleotide calls from population-based sequencing of HIV-1 are a marker for viral diversity and the age of infection. Clin Infect Dis 52: 532.

2. Ragonnet-Cronin M, Aris-Brosou S, Joanisse I, Merks H, Vallée D, et al. (2012) Genetic diversity as a marker for timing infection in HIV-infected patients: Evaluation of a 6-month window and comparison with BED. J Infect Dis 206: 756–764.

3. Taffé P, May M (2008) A joint back calculation model for the imputation of the date of HIV infection in a prevalent cohort. Stat Med 27: 4835–4853.

4. Hand DJ, Yu K (2001) Idiot's BayesNot so stupid after all? International Statistical Review 69: 385–398.

5. Volz E, Pond S, Ward M, Leigh Brown A, Frost S (2009) Phylodynamics of infectious disease epidemics. Genetics 183: 1421–1430.

6. Frost S, Volz E (2010) Viral phylodynamics and the search for an effective number of infections. Philos Trans R Soc Lond B Biol Sci 365: 1879.

7. Rasmussen D, Ratmann O, Koelle K (2011) Inference for nonlinear epidemiological models using genealogies and time series. PLoS Comput Biol 7: e1002136.

8. Koelle K, Rasmussen D (2012) Rates of coalescence for common epidemiological models at equilibrium. Journal of The Royal Society Interface 9: 997–1007.

9. Volz E (2012) Complex population dynamics and the coalescent under neutrality. Genetics 190: 187–201.

10. Efron B (2010) Large-scale Inference: Empirical Bayes Methods for Estimation, Testing and Prediction. Cambridge.

11. Ayres D, Darling A, Zwickl D, Beerli P, Holder M, et al. (2012) Beagle: an application programming interface and high-performance computing library for statistical phylogenetics. Systematic Biology 61: 170–173.
